# Teaching Model Context Protocol, Retrieval-Augmented Generation, and AI Agents to a Multidisciplinary Hospital Workforce: Single-Group Pre-Post Survey Study

**DOI:** 10.2196/97822

**Published:** 2026-09-11

**Authors:** Gakyoung Baek, Hyunna Lee, Dong Hyun Yang, Minseo Kang, Kun Hee Lee, Minji Choi, Yura Lee, Kye Hwa Lee

**Affiliations:** 1 Big Data Research Center, Asan Institute for Life Sciences, Asan Medical Center, Seoul, Republic of Korea; 2 Department of Radiology and Research Institute of Radiology, University of Ulsan College of Medicine, Asan Medical Center, Seoul, Republic of Korea; 3 Department of Information Medicine, Asan Medical Center, University of Ulsan College of Medicine, 88 Olympic-ro 43-gil, Songpa-gu, Seoul, 05505, Republic of Korea, 82 2-3010-5991

**Keywords:** artificial intelligence, medical education, large language models, self-efficacy, health personnel, model context protocol, staff development, curriculum design

## Abstract

**Background:**

Hospitals worldwide need to upskill their workforce in advanced AI technologies; yet, published guidance on how to design and deliver such training, particularly in agent-level tools like retrieval-augmented generation (RAG) and the model context protocol (MCP), remains virtually absent.

**Objective:**

To describe the design, implementation, and lessons learned from an 8-week, 56-hour intensive generative AI training program for a multidisciplinary hospital workforce, drawing on both quantitative outcome data and participants’ own reflections on their learning experience.

**Methods:**

The program was delivered on-site at Asan Medical Center with simultaneous online broadcast to 2 regional affiliate hospitals. The curriculum was built around the premise that MCP and AI agents would become the foundation of health care AI use, allocating 37% (11.5/31 hours) of on-site instructional time to MCP, and 71% (22/31 hours) to hands-on practice. Participants progressed from foundational concepts through RAG and MCP to team-based capstone projects, supported by funded AI tool subscriptions, a dedicated internal cloud platform, and 3‐6 hours of weekly mentoring per team. A pre-post survey (pre: n=83; post: n=64) evaluated outcomes across Kirkpatrick levels 1‐3, complemented by thematic analysis of open-ended reflections on self-perceived growth.

**Results:**

The technologies that received the greatest curricular investment were associated with the largest self-efficacy differences (MCP: *d*=1.57; overall effect: *r*=.574), and participants most frequently cited MCP and RAG when describing how abstract concepts “became concrete and actionable.” Non-IT professionals, clinicians, health information managers, researchers, and administrative staff showed consistently larger gains than IT specialists; several reported coding for the first time through vibe coding, challenging the assumption that advanced AI training requires technical backgrounds. Despite significant overall gains, a knowledge-practice gap persisted: job-specific competency remained below the scale midpoint, though participants spontaneously reported generating workplace application ideas. Curriculum pacing was rated lowest despite high overall satisfaction (4.03/5), signaling that even 56 hours may progress too quickly for mixed-expertise cohorts. Capstone projects with dedicated mentoring received the highest satisfaction ratings; 11 of 12 teams presented functional prototypes, and one has since entered active pilot use in clinical departments ahead of planned hospital-wide deployment.

**Conclusions:**

To our knowledge, this is the first program to teach 4 agent-level generative AI technologies, MCP, RAG, LangGraph orchestration, and AI agent design, to both IT and non-IT hospital staff. This program suggests that transforming a multidisciplinary hospital workforce into AI-capable professionals is achievable through intensive, hands-on training centered on agent-level technologies, and that capstone projects with dedicated mentoring can serve as a pathway from classroom learning toward institutional AI adoption. The knowledge-practice gap highlights the need for posttraining support structures to translate self-efficacy gains into sustained workplace practice.

## Introduction

### Background

Hospitals that want to harness AI for clinical documentation, decision support, and administrative automation [1-4] face a workforce problem: the technologies that make these applications possible, retrieval-augmented generation (RAG) [5], the model context protocol (MCP) [6], and orchestration frameworks such as LangGraph [7], are evolving faster than the people who must build, adapt, and evaluate them [8,9]. The question is no longer whether to train hospital staff in advanced AI, but how.

Existing training efforts offer limited guidance. The 2024 Best Evidence Medical Education (BEME) scoping review by Gordon et al [10] confirmed that published AI education programs remain predominantly foundational—covering AI literacy, prompt engineering, and learners’ perceptions of generative AI tools such as ChatGPT [11-13]—and target mainly medical students and residents [14,15]. Evidence on intensive, multiweek programs that teach advanced technologies to the broader hospital workforce—IT specialists, health information managers (HIM), clinicians, researchers, and administrative staff—is scarce [16-18]. No published study, to our knowledge, has reported the design and outcomes of training that covers RAG, MCP, AI agent design, and workflow orchestration together.

### Prior Work

Self-efficacy theory [19] predicts that mastery experiences, particularly hands-on practice with novel technologies, are the strongest driver of sustained technology adoption [20-22]. Consistent with this, recent ChatGPT education studies report that structured training significantly raises perceived AI competency among health care professionals [8,12], and experiential learning approaches outperform didactic instruction for skill-based outcomes [23]. However, most evaluations remain limited to satisfaction surveys (Kirkpatrick level 1 [24,25]), with few assessing knowledge gains (Level 2) or behavioral transfer intentions (Level 3) [26,27]. Our previous evaluation of a health informatics analyst program at the same institution demonstrated that intensive education can reshape job roles and skill profiles [18]; this study extends that work to advanced generative AI technologies.

### Study Objectives

This study has two complementary aims: (1) to describe the design rationale, curriculum structure, and implementation experience of an 8-week, 56-hour intensive generative AI training program for a multidisciplinary hospital workforce at a tertiary academic medical center; and (2) to evaluate its outcomes using the Kirkpatrick framework across Levels 1‐3, providing empirical evidence that contextualizes the lessons learned. We addressed four research questions (RQs):

1. RQ1: Are posttraining AI knowledge self-efficacy and job-specific AI competency scores higher than pretraining scores? (Level 2; S4, S6)
2. RQ2: How satisfied are participants with the training program, and which curricular components are rated highest and lowest? (Level 1; S9)
3. RQ3: What are participants’ behavioral intentions to apply learned skills, and where do gaps between knowledge and application emerge? (Level 3 proxy; S11)
4. RQ4: Do between-group differences in AI knowledge self-efficacy vary by professional group, and what does this imply for participant selection?

## Methods

### Study Design

This was a single-group, pre-post survey study using an independent samples design conducted at a single tertiary academic medical center in Seoul, South Korea. The training program was administered from September to November 2025, with surveys distributed immediately before (pretraining) and after (posttraining) the program. Because survey responses were collected anonymously without personal identifiers, individual-level matching between pre- and posttraining responses was not possible; therefore, pre- and posttraining groups were treated as independent samples. This study adhered to the STROBE (Strengthening the Reporting of Observational Studies in Epidemiology) guidelines for cross-sectional studies [28].

### Participants

Eligible participants were employees of Asan Medical Center, Ulsan University Hospital, or Gangneung Asan Hospital who enrolled in the advanced generative AI training program. The program was delivered on-site at Asan Medical Center with simultaneous online broadcast to the 2 regional affiliate hospitals, enabling multisite participation. Inclusion criteria were (1) current employment at one of the participating institutions, (2) voluntary enrollment in the program, and (3) age 18 years or older. Participants who attended fewer than 80% of the total training hours were excluded; all 83 enrollees met this threshold. Recruitment did not use an open call; instead, the program was promoted internally within candidate departments through departmental announcements and circulated notices, and interested employees enrolled voluntarily. Because the program’s primary aim was to build AI-agent development capability, enrollment was prioritized in the order of IT workforce, health-information staff, clinicians, and researchers, with IT and related departments approached first.

All 83 enrollees completed the pretraining survey. Of these, 64 responded to the posttraining survey (response rate: 77.1%). A total of 147 survey responses were thus analyzed (pre: n=83; post: n=64). Demographic and occupational characteristics, professional group and years of work experience, were self-reported by participants as part of both the pre- and posttraining surveys, without collection of personal identifiers, and are detailed in the Results section and Table 1.

**Table 1.** Participant demographics.

Characteristics	Pretraining (n=83)	Posttraining (n=64)	*P* value
Occupation, n (%)			.87^a^
IT specialists	48 (57.8)	33 (51.6)	
HIM^b^	10 (12)	9 (14.1)	
Clinicians	9 (10.8)	7 (10.9)	
Researchers	9 (10.8)	8 (12.5)	
Administrative staff	7 (8.4)	7 (10.9)	
Work experience, n (%)			.85^a^
<1 year	3 (3.6)	3 (4.7)	
1‐5 years	13 (15.7)	12 (18.8)	
6‐10 years	18 (21.7)	14 (21.9)	
≥11 years	49 (59.0)	35 (54.7)	

a chi-squared test.

b HIM: health information managers.

### Training Program: Design Rationale and Curriculum Structure

#### Overview

The training program was conducted as part of the Medical AI Healthcare Professional Continuing Education Project, a national initiative organized by the Ministry of Health and Welfare and the Korea Human Resource Development Institute for Health & Welfare (KOHI). This institutional framework provided the mandate and funding structure for delivering advanced AI training to hospital staff, and situates the present program within a broader national effort to build health care AI workforce capacity.

#### Design Philosophy

Within this framework, the program was designed around a central premise: that MCP and AI agents would become the foundational infrastructure for health care AI use, much as databases became foundational for health informatics. Rather than teaching AI as a collection of isolated tools, the curriculum treated agent-level capabilities, connecting AI models to institutional data sources, orchestrating multistep workflows, and building domain-specific applications, as the core competency target. This premise motivated two key design decisions: (1) allocating the largest share of instructional time to MCP (11.5 h, 37% of instructional time), and (2) requiring all participants to build functional MCP-based prototypes through team capstone projects, on the reasoning that hands-on development experience is the fastest path to genuine understanding and use of AI agent architectures. Relatedly, the inaugural cohort was deliberately composed of staff positioned to enable hospital-wide AI adoption, IT and health-information personnel, related-department clinicians, and researchers, as the first phase of a staged institutional strategy rather than as an end in itself. This IT-first prioritization reflected an assumption that the interface between clinicians and IT professionals will expand substantially in the AI-agent era; building hospital-needs-driven agent-development capability first was expected to lay the foundation for later, clinician-needs-driven AI adoption.

The program prioritized experiential learning. Hands-on practice comprised 71% of on-site instructional time (22 of 31 h); the remaining 9 hours (29%) were didactic lectures. The subsequent 3-week team capstone added 25 hours of project-based work, so experiential learning dominated the 56-hour program overall (47 of 56 h, 84%). This ratio was informed by self-efficacy theory [19], which predicts that mastery experiences—direct, successful practice—are the most potent source of self-efficacy, and by evidence that experiential learning produces stronger skill-based outcomes than didactic instruction alone [23]. Because the cohort spanned a wide range of baseline technical skill—from health-information staff who had never written code to experienced IT specialists—the curriculum also deliberately minimized AI theory (such as the mathematics of deep learning or the internal mechanics of large language models [LLMs] and agents), emphasizing instead practical terminology, core concepts, the essential agentic methods participants needed, and concrete MCP use cases and development techniques. Within the hands-on sessions, participants worked in 2 parallel tracks matched to baseline skill—an application track (assembling solutions with existing frameworks, such as a RAG pipeline in LangChain) and a development track (implementing components directly, such as embedding and similarity search)—so that both first-time coders and experienced developers were appropriately challenged.

To ensure that all participants could engage in hands-on practice without technical barriers, the program provided substantial infrastructure support. Each participant received 3-month funded subscriptions to commercial AI development tools (Cursor IDE [Anysphere], Claude API, and GPT API), removing cost as an obstacle to experimentation. Additionally, the institution deployed a dedicated internal cloud environment where participants could design, test, and run MCP servers using local LLMs within the hospital’s secure network, addressing the data governance constraints that typically impede AI development in health care settings.

#### Curriculum Structure

The 8-week, 56-hour program consisted of 5 on-site instructional sessions (31 h) and a 3-week team-based capstone project (25 h; Table S1 in Multimedia Appendix 1). The curriculum followed a scaffolded progression designed to build competencies incrementally:

1. Week 1 — foundations (7 h): foundation models and prompt engineering. Established shared vocabulary and baseline skills across all professional groups.
2. Week 2 — RAG and LangGraph workflow design (7 h): RAG [5] and medical document question-answering systems. Introduced the concept of grounding AI outputs in institutional knowledge bases. In the hands-on session, participants built a working RAG pipeline that answered questions over a corpus of institutional clinical documents. The afternoon introduced LangGraph for graph-based clinical-workflow design, modeling a care process (registration → triage → testing → diagnosis → prescription) as nodes and edges.
3. Week 3 — MCP and vector databases (7 h): MCP [6] fundamentals and vector database integration. As a hands-on exercise, participants built their first MCP server and connected it to a vector database of internal guidelines.
4. Week 4 — advanced agent design (7 h): advanced MCP server development and LangGraph-based AI agent workflows [7,29]. As capstone preparation, participants designed a multistep, tool-calling clinical workflow agent in LangGraph.
5. Weeks 5‐7 — team capstone projects (25 h): Self-directed team projects in which participants developed working AI prototypes for real health care challenges. Teams were composed of mixed professional backgrounds (IT specialists, clinicians, HIMs, researchers, and administrative staff) to leverage domain expertise alongside technical skills.
6. Week 8 — integration (3 h): Advanced MCP host development and workflow integration; the program concluded with capstone presentations and an awards ceremony.

The technology-specific instructional hours, calculated as a percentage of the 31 on-site instructional hours, were distributed as follows: MCP (11.5 h, 37%), LangGraph workflow design (8.5 h, 27%), foundation models and prompt engineering (7 h, 23%), RAG (5 h, 16%), medical Question and Answer (Q&A) systems (5 h, 16%), and AI agent design (4 h, 13%). Because some sessions covered more than one technology, their hours were counted in each relevant category, so the listed percentages sum to more than 100%. The heavy MCP allocation reflected the program’s core thesis that agent-level infrastructure would be the most transferable and enduring competency.

A detailed session-level syllabus, including per-session learning objectives, hands-on exercises, and tools for all 8 weeks, is provided in Multimedia Appendix 2. To support reproducibility while respecting institutional and funding constraints, we release this syllabus and the capstone evaluation rubric (Multimedia Appendix 3); full scaffold code, proprietary MCP server templates, and internal datasets are not released owing to institutional intellectual-property policy and national-program KOHI constraints.

#### Capstone Projects

All participants were organized into 12 teams, including one team each from Ulsan University Hospital and Gangneung Asan Hospital, each comprising a mix of professional backgrounds. To preserve familiar communication channels while combining expertise, each team was built around an intact IT subdepartment as its technical core, augmented with non-IT domain members (for example, an IT infrastructure team joined by 2 health-information staff and a clinician); this structure was applied consistently across all teams. Each team identified a real-world health care problem within their work context and developed a functional MCP-based AI prototype addressing it over 3 weeks. A single dedicated mentor, selected from the program instructors, provided 3‐6 hours of structured mentoring per team per week throughout the capstone period, ensuring consistent guidance across all projects. The capstone served dual purposes: providing extended development time for skill consolidation and generating tangible evidence of practical competency beyond self-report measures.

Capstone projects were evaluated at the closing ceremony (November 11, 2025) by a panel of 7 assessors using a standardized 100-point rubric comprising four weighted domains—AI technical use (40 points; 4 items), user experience and user interface (20 points; 2 items), completeness and stability (20 points; 2 items), and clinical applicability (20 points; 2 items)—with each item rated on a 1‐10 scale. To adjust for differences in scoring stringency across assessors, each assessor’s raw scores were standardized (z-transformed) within assessor, converted to rank-based scores, and aggregated across the 7 assessors to determine final standings. The full rubric is provided in Multimedia Appendix 3.

### Outcome Measures

Survey items were developed by the research team specifically for this program, grounded in the competency domains of the training curriculum (MCP, RAG, LangGraph, AI agent design, and domain-specific applications) and informed by Compeau and Higgins [20] computer self-efficacy scale, adapted to the generative AI domain. The instrument underwent internal review by the program instructors prior to deployment. Post hoc psychometric analyses (Cronbach α, item-total correlations, exploratory factor analysis [EFA]) are reported in Tables S5-S6 in Multimedia Appendix 1.

Survey instruments consisted of 100 pretraining items and 154 posttraining items organized into 11 sections. All attitudinal and self-efficacy items used a 5-point Likert scale (1=strongly disagree to 5=strongly agree). The measures were mapped to the Kirkpatrick 4-level training evaluation model [24,25] as follows:

Primary outcomes (pre-post comparison):

1. AI knowledge self-efficacy (S4; 8 items): self-assessed competency across 8 AI domains, basic AI understanding, prompt engineering, RAG, local LLM deployment, LangGraph workflow, MCP, medical Q&A systems, and AI agent design. This corresponded to Kirkpatrick level 2 (learning).
2. Job-specific AI competency (S6; 7 items per professional group): self-assessed ability to apply AI tools to job-specific tasks, corresponding to Kirkpatrick level 2. Although each professional group received items tailored to their work context, all items assessed the same underlying construct (perceived ability to apply AI to professional tasks) on an identical response scale, enabling aggregation for overall pre-post comparisons.

Secondary outcomes (pre-post comparison):

1. AI attitudes (S2; 9 items): perceptions toward AI adoption, including apprehension, replacement concerns, and adaptation confidence.
2. AI daily usage (S3; 2 categorical items): frequency and duration of AI tool use in daily work.
3. Digital and programming competency (S5; 4 Likert items for digital competency, plus multiselect programming language proficiency): self-assessed digital skills and programming ability. Scale analysis was based on the 4 digital competency items.

Posttraining only outcomes:

1. Training satisfaction (S9; 18 items across 6 domains): curriculum design, content and practice, instructor quality, teaching methods, team project experience, and learning environment, corresponding to Kirkpatrick level 1 (reaction).
2. Perceived changes (S10; 10 items): self-reported changes in knowledge, skills, attitudes, and workplace application confidence following training.
3. Workplace application intention (S11; 6 core Likert items + supplementary topic-specific and categorical items): plans for applying learned skills to current work, peer sharing, and continued learning, corresponding to Kirkpatrick level 3 (behavioral intention). S11 measures behavioral intention rather than observed behavioral change; true level 3 assessment would require longitudinal follow-up, which was beyond the scope of this study. Scale analysis was based on the 6 core items.
4. Self-perceived growth (1 open-ended item): “What aspect of change or growth did you most strongly feel through this 8-week program?” All 64 responses were read in full by the research team. Recurring themes were identified inductively from the responses rather than applied from an a priori framework, and a set of 5 themes was agreed upon. Each response was then assigned to 1 or 2 of these themes (responses too brief to interpret were set aside as unclassifiable), and the number of responses per theme was tallied. The responses were originally written in Korean; representative quotations were translated into English, and the complete set of translated responses is provided in Multimedia Appendix 4.

### Statistical Analysis

All analyses were performed using Python 3.x (pandas v2.0, SciPy v1.11, factor_analyzer). The anonymous design required treating pre- and posttraining groups as independent samples. Mann-Whitney *U* tests [30] were used for pre-post comparisons, with the rank-biserial correlation *r* (= |Z| / √N; Rosenthal [31]) as the primary effect size, interpreted as small (≥.10), medium (≥.30), or large (≥.50) per Cohen [32]. Cohen *d* was additionally reported to facilitate comparison with prior literature. The significance threshold was α=0.05 (2-tailed); posttraining-only measures (S9, S10, and S11) were summarized descriptively. Chi-squared tests assessed demographic comparability between the 2 independent samples.

Subgroup analyses by professional group were exploratory: Cohen *d* with 95% CIs was computed without formal hypothesis testing or multiple-comparisons correction, given small cell sizes (n=7‐9 for non-IT groups). Full inferential statistics are available in Table S2 in Multimedia Appendix 1.

Internal consistency was assessed using Cronbach α (acceptable≥0.70, good≥0.80 [33]). EFA (principal axis factoring, direct oblimin rotation) and corrected item-total correlations provided post hoc construct validity evidence (Tables S5-S6 in Multimedia Appendix 1).

A worst-case sensitivity analysis assigned all 19 nonrespondents’ posttraining scores equal to their professional group’s pretraining mean (Table S7 in Multimedia Appendix 1). A post hoc power analysis indicated the minimum detectable effect at 80% power was *r*=.24 (Table S4 in Multimedia Appendix 1).

### Ethical Considerations

The study protocol was approved by the Institutional Review Board of Asan Medical Center (IRB number 2025‐1070; approved August 28, 2025). Written informed consent was waived because data were collected anonymously through voluntary online surveys, participation posed no more than minimal risk, and the study could not practicably be conducted without the waiver. The first page of the survey informed participants of the study purpose, voluntary nature, and data handling procedures. Participants received no monetary compensation; program tuition was fully funded through the national continuing-education initiative administered by the KOHI.

## Results

### Participant Characteristics

**Figure 1.**
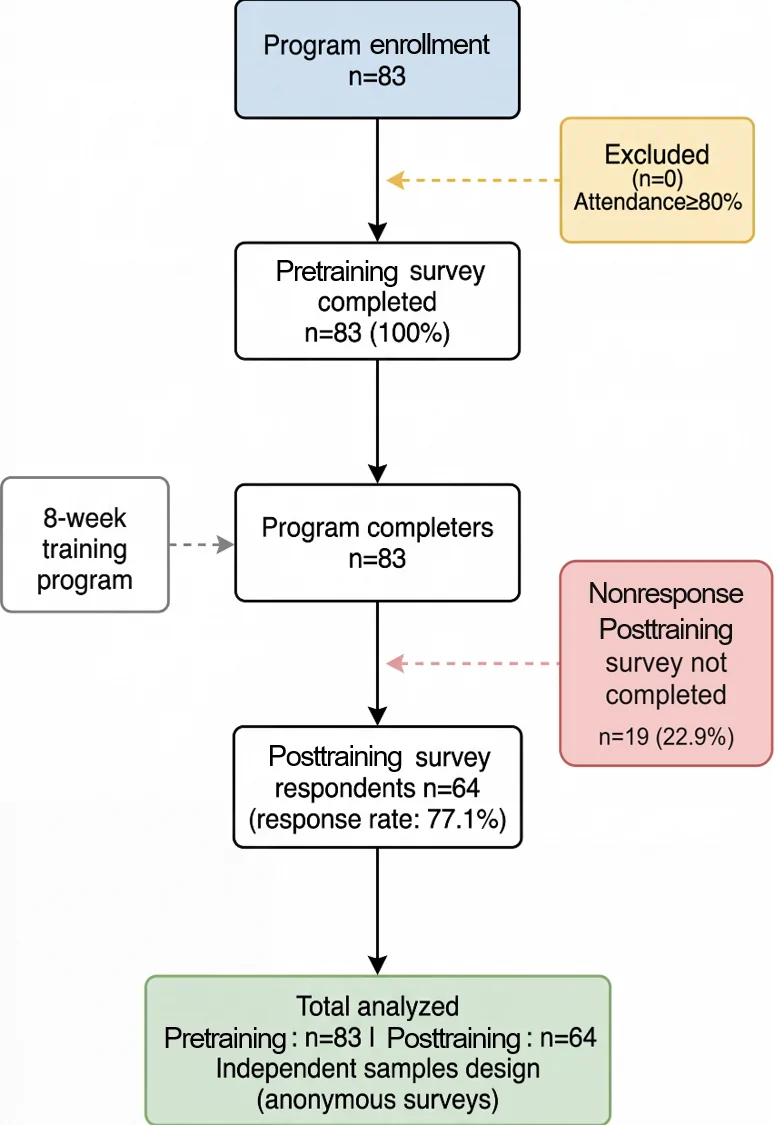
Participant flow diagram showing enrollment (n=83), completion of the 8-week training program (n=83), and response rates for the pretraining (n=83; 100%) and posttraining (n=64; 77.1%) surveys. No participants were excluded, as all enrollees met the ≥80% attendance criterion. Because the surveys were anonymous, the pretraining and posttraining groups were analyzed as independent samples.

All 83 enrollees completed the program and responded to the pretraining survey; 64 responded to the posttraining survey (response rate: 77.1%; pre: n=83; post: n=64; Figure 1). Demographic characteristics of the pre- and posttraining groups are presented in Table 1. The occupational distribution was comparable between the 2 groups (IT specialists: 57.8%, 48/83 pre vs 51.6%, 33/64 post; HIM: 12%, 10/83, vs 14.1%, 9/64; clinicians: 10.8%, 9/83, vs 10.9%, 7/64; researchers: 10.8%, 9/83, vs 12.5%, 8/64; administrative staff: 8.4%, 7/83, vs 10.9%, 7/64); retention was lower among IT specialists (33/48, 68.8%) than among non-IT groups (78%, 7/9 to 100%, 7/7; see Limitations). The majority of participants in both groups had over 11 years of experience (59%, 49/83 pre vs 54.7%, 35/64 post). Chi-squared tests confirmed no statistically significant differences in occupational (*χ*²_4_=1.23; *P*=.87) or experience-level (*χ*²_3_=0.80; *P*=.85) distributions between the 2 independent samples.

### Primary Outcomes: AI Knowledge Self-Efficacy (S4)

**Figure 2.**
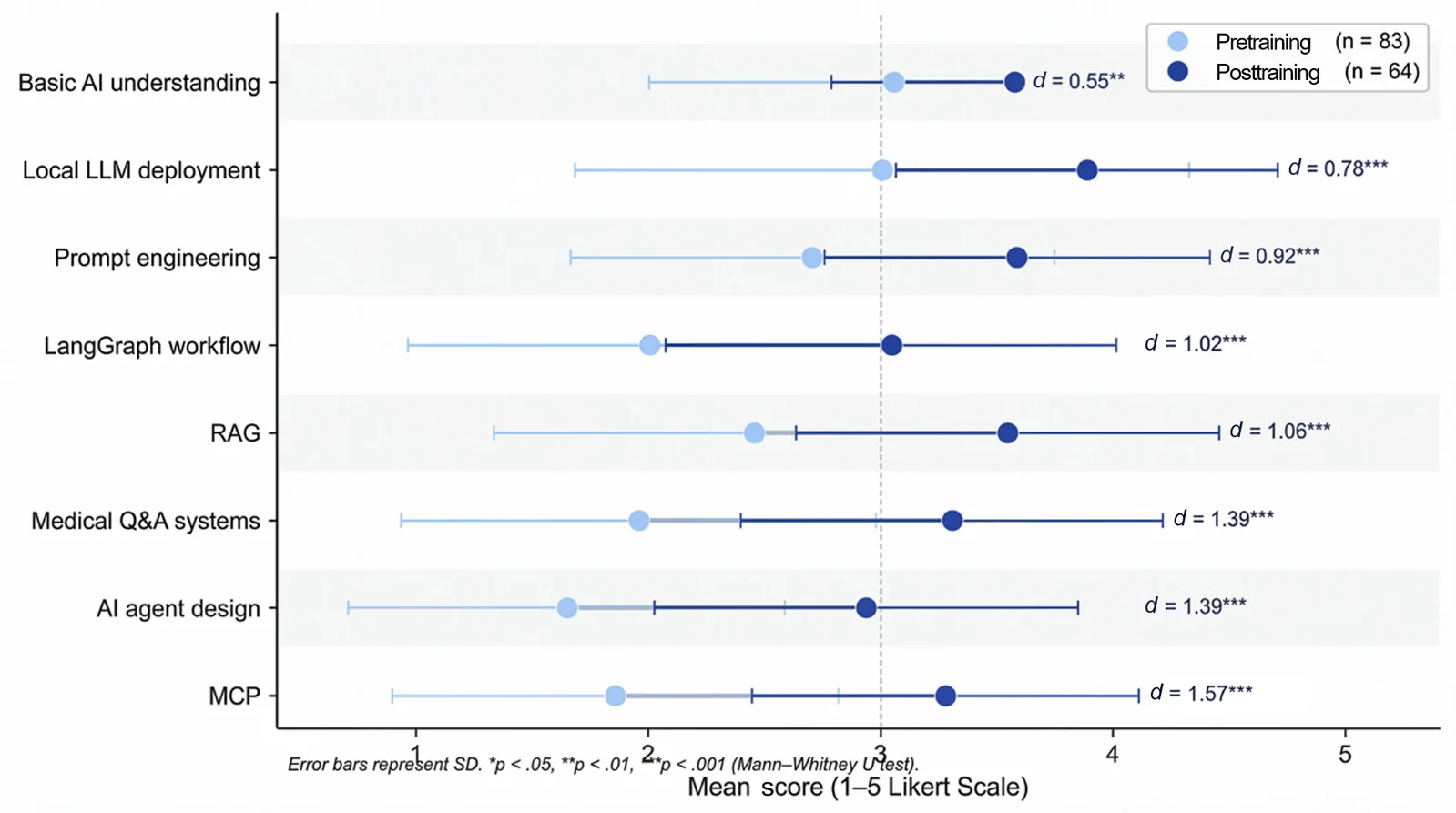
Comparison of self-assessed AI competency scores across eight skill domains before (n=83) and after (n=64) the training program, with Cohen *d* effect sizes and Mann-Whitney *U* test significance levels. MCP: model context protocol; LLM: large-language model; RAG: retrieval-augmented generation.

Posttraining AI knowledge self-efficacy scores were significantly higher than pretraining scores (mean 3.40, SD 0.69 vs mean 2.34, SD 0.83; *U*=875.5, *r*=.574, *d*=1.37; *P*=3.50×10^–^¹²; Table 2, Figure 2). Internal consistency was excellent (α=0.909 pre, α=0.915 post; Tables S5-S6 in Multimedia Appendix 1). At the domain level, the technologies that received the greatest curricular investment and had the lowest baseline scores showed the largest between-group differences: MCP (*d*=1.57), medical Q&A systems (*d*=1.39), and AI agent design (*d*=1.39), whereas foundational domains with higher baselines showed smaller differences (basic AI understanding: *d*=0.55; Table 2).

**Table 2.** Pre-Post comparison of primary and secondary outcomes. r=rank-biserial correlation (Rosenthal formula); significance threshold: α=0.05.

Measures	Pre mean (SD)	Post mean (SD)	*U*	*Z*	*P*	*r*	Cohen *d*	Interpretation
S4: AI knowledge self-efficacy	2.34 (0.83)	3.40 (0.69)	875.5	–6.956	3.50×10^–^¹²	.574	1.37	Large
S6: Job-specific AI competency	1.95 (1.00)	2.77 (0.95)	1470	–4.634	3.59×10^–^⁶	.382	0.85	Medium
S2: AI attitudes	3.79 (0.41)	3.77 (0.51)	2733	–0.301	.764	.025	–0.04	Negligible
S5: Digital competency	3.38 (0.89)	3.63 (0.82)	2228.5	–1.675	.094	.138	0.29	Small

### Subgroup Analysis by Professional Group

All 5 professional groups showed the largest between-group difference in MCP, and non-IT groups consistently showed larger differences than IT specialists (Table S2 in Multimedia Appendix 1). For MCP, clinicians showed the largest difference (*d*=2.88, 95% CI 1.42-4.33), followed by HIM (*d*=2.45, 95% CI 1.23-3.67), researchers (*d*=2.07, 95% CI 0.87-3.28), and administrative staff (*d*=1.83, 95% CI 0.55-3.11); IT specialists showed the smallest difference (*d*=1.36, 95% CI 0.87-1.85). These subgroup findings should be considered exploratory given small cell sizes (n=7‐9 for non-IT groups; Table S2 in Multimedia Appendix 1).

### Secondary Outcomes and the Knowledge-Practice Gap

Job-specific AI competency (S6) scores were significantly higher posttraining (mean 2.77, SD 0.95 vs mean 1.95, SD 1; *P*=3.59×10^–^⁶, *r*=.382, *d*=0.85; α=0.938-0.973 pre, α=0.877-0.981 post; Table 2). However, unlike general AI self-efficacy (S4), job-specific competency remained below the scale midpoint (2.77/5.0), indicating a knowledge-practice gap.

Three secondary pre-post comparisons did not reach significance (Table 2; Table S4 in Multimedia Appendix 1): AI attitudes (S2: *r*=.025; *P*=.76), with the mean of the 6 positively worded items (Q4-Q9) already at ceiling at baseline (M=4.29/5); the full 9-item S2 scale mean, with reverse coding applied to Q1-Q3, was 3.79 (Table 2); digital competency (S5: *r*=.138; *P*=.09); and AI daily usage (S3), where the proportion using 3+ hours daily increased modestly from 9.6% to 17.2%.

### Training Satisfaction (S9; Kirkpatrick Level 1)

**Figure 3.**
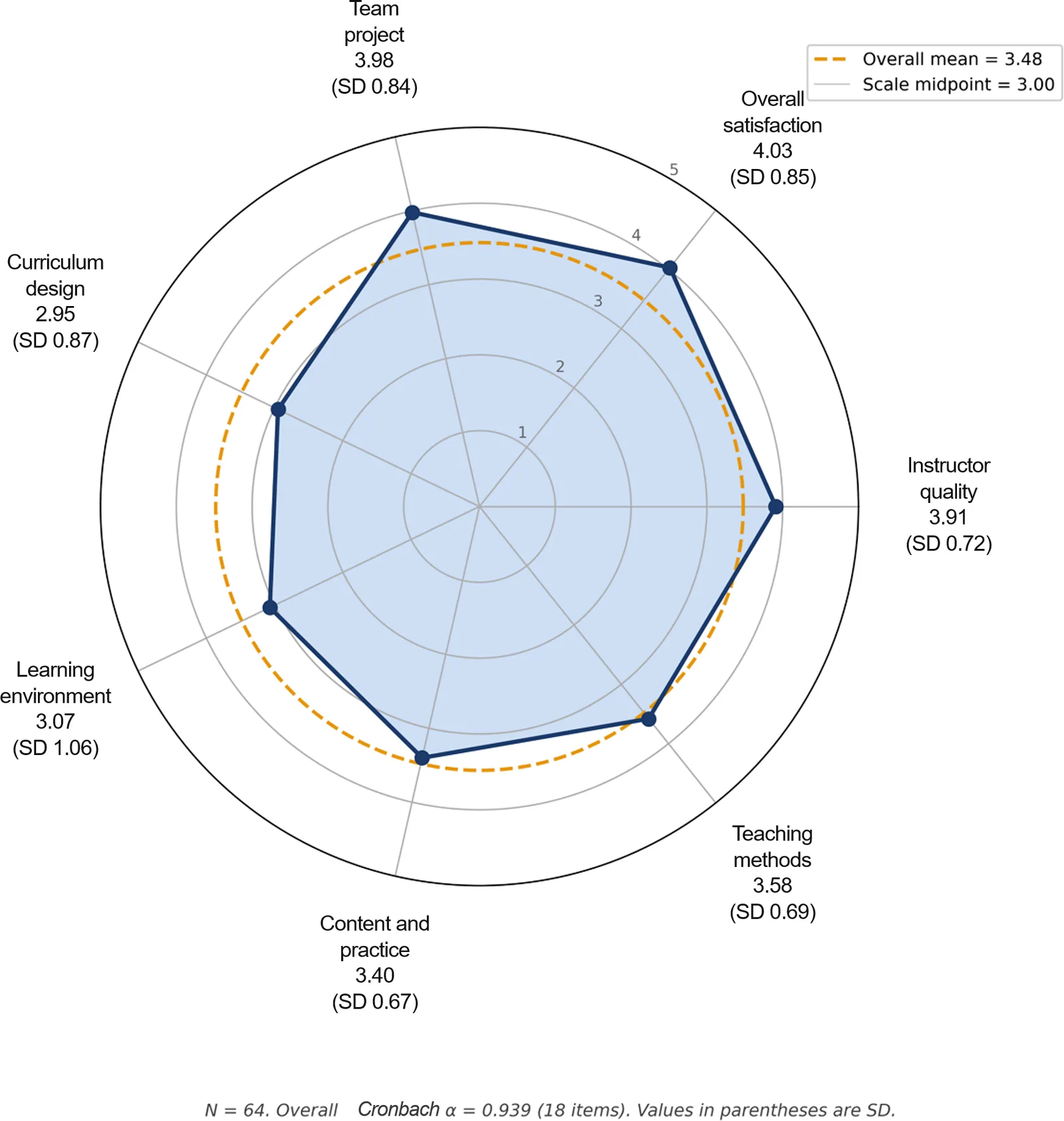
Radar chart of program satisfaction ratings across seven evaluation dimensions (N=64; overall Cronbach α=0.939, 18 items), where the dashed orange line represents the overall mean (3.48) and the gray dashed circle indicates the scale midpoint (3).

Overall training satisfaction was 4.03/5.0 (SD 0.85), while the domain-averaged score was 3.48 (SD 0.63; α=0.939; Table 3, Figure 3). Satisfaction varied substantially across domains: team project experience was rated highest (mean 3.98, SD 0.84), followed by instructor quality (mean 3.91, SD 0.72), teaching methods (mean 3.58, SD 0.69), and content & practice (mean 3.40, SD 0.67). Curriculum design was rated lowest (mean 2.95, SD 0.87), particularly learning pace (mean 2.91, SD 1.11). Item-level results for all posttraining scales (S9-S11) are reported in Table S3 in Multimedia Appendix 1.

**Table 3.** Training satisfaction by domain (S9).

Domain	Mean (SD)	Cronbach α
Team project experience	3.98 (0.84)	0.789
Instructor quality	3.91 (0.72)	0.857
Teaching methods	3.58 (0.69)	0.833
Content and practice	3.40 (0.67)	0.845
Learning environment	3.07 (1.06)	0.912
Curriculum design	2.95 (0.87)	0.867
Overall (single item)	4.03 (0.85)	—^a^
Domain average	3.48 (0.63)	0.939

a Not available.

### Capstone Outcomes and Real-World Deployment

Of the 12 capstone teams, 11 presented functional AI prototypes spanning clinical, administrative, and IT applications (representative examples in Table 4; full list in Table S8 in Multimedia Appendix 1); one regional affiliate team was unable to present due to scheduling constraints. One project, a medical dictionary-based RAG/query chatbot system (“WorksBot”), received the Minister of Health and Welfare Award. One prototype, the hospital document knowledge retrieval RAG system, has since been developed into an institutional service (“AI-Docs”) and has been in active pilot use since May 2026 across multiple departments, including the Department of Nursing and the Big Data Research Center, with hospital-wide deployment planned within 2026. WorksBot remains a prototype and is not yet in operational service; a refined version is planned following the RAG pilot. Both efforts are being advanced by the Department of Digital Innovation and Support.

**Table 4.** Representative capstone projects. Representative projects illustrating the range of clinical, operational, and IT applications; the complete list of all 12 capstone projects is provided in Table S8 in Multimedia Appendix 1.

Projects	Domain	Core technologies	Status
WorksBot (medical-dictionary RAG^a^/ or Q&A)^b^	Clinical knowledge access	MCP^c^, RAG, vector DB^d^	Top-ranked; prototype
AI-Docs (document-retrieval RAG)	Hospital operations	MCP, RAG, vector DB	Active pilot; hospital-wide planned 2026
Natural-language clinical-data retrieval	Clinical	MCP, LangGraph	Prototype
Medical-record de-identification	Data governance or IT	MCP, local LLM^e^	Prototype
AGS^f^ operational-guideline Q&A chatbot	Hospital operations	MCP, RAG, local LLM	Prototype

a RAG: retrieval-augmented generation.

b Q&A: question and answer.

c MCP: model context protocol.

d Vector DB: vector database.

e LLM: large-language model.

f AGS: Asan Global Standard, the institution's internal operational guideline system.

### Posttraining Outcomes (S10, S11; Kirkpatrick Levels 2-3)

**Figure 4.**
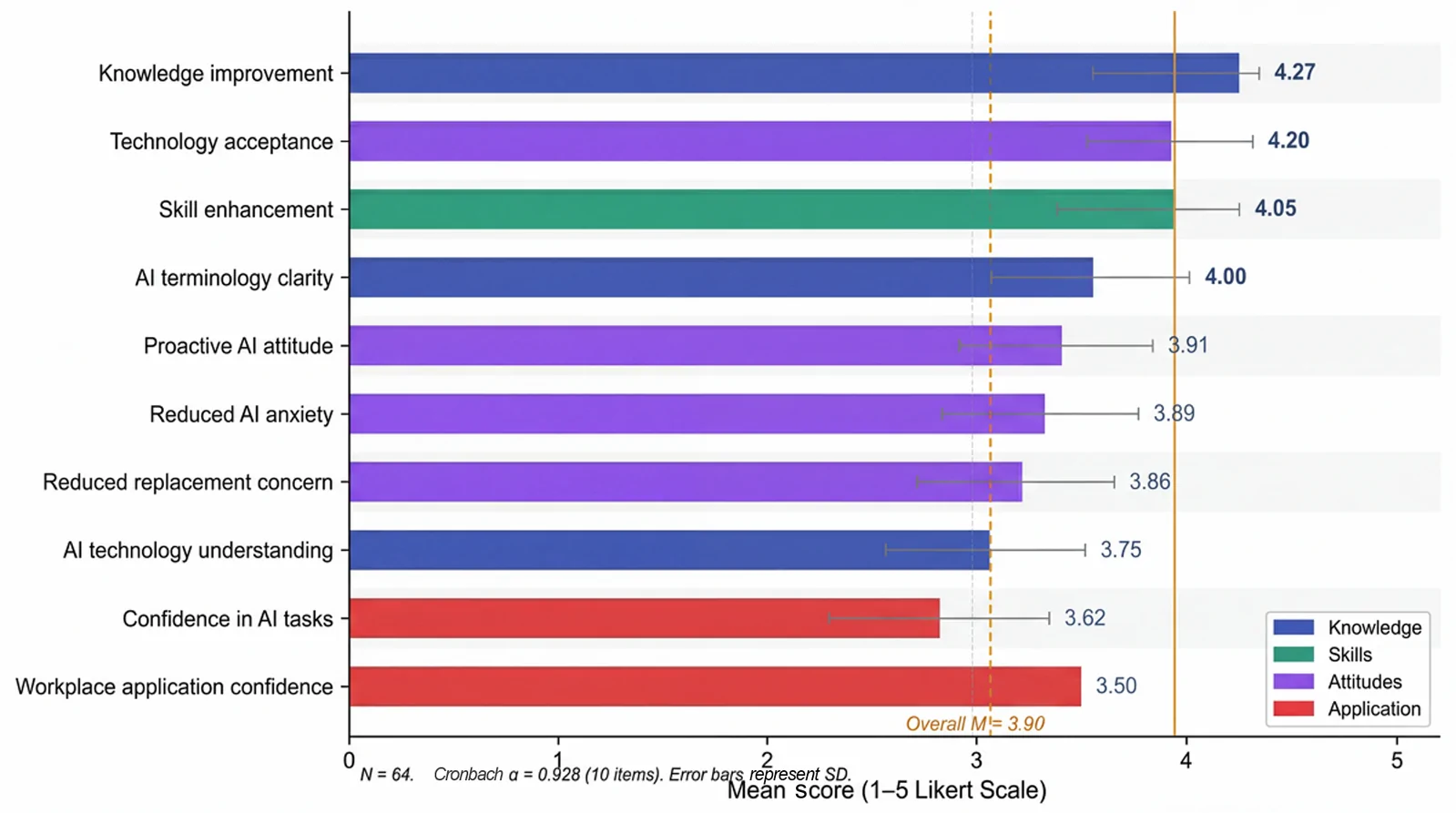
Self-perceived educational effectiveness across 10 items categorized into knowledge, skills, attitudes, and application domains (N=64; Cronbach α=0.928, 10 items), with an overall mean of 3.90 and error bars representing standard deviations.

**Figure 5.**
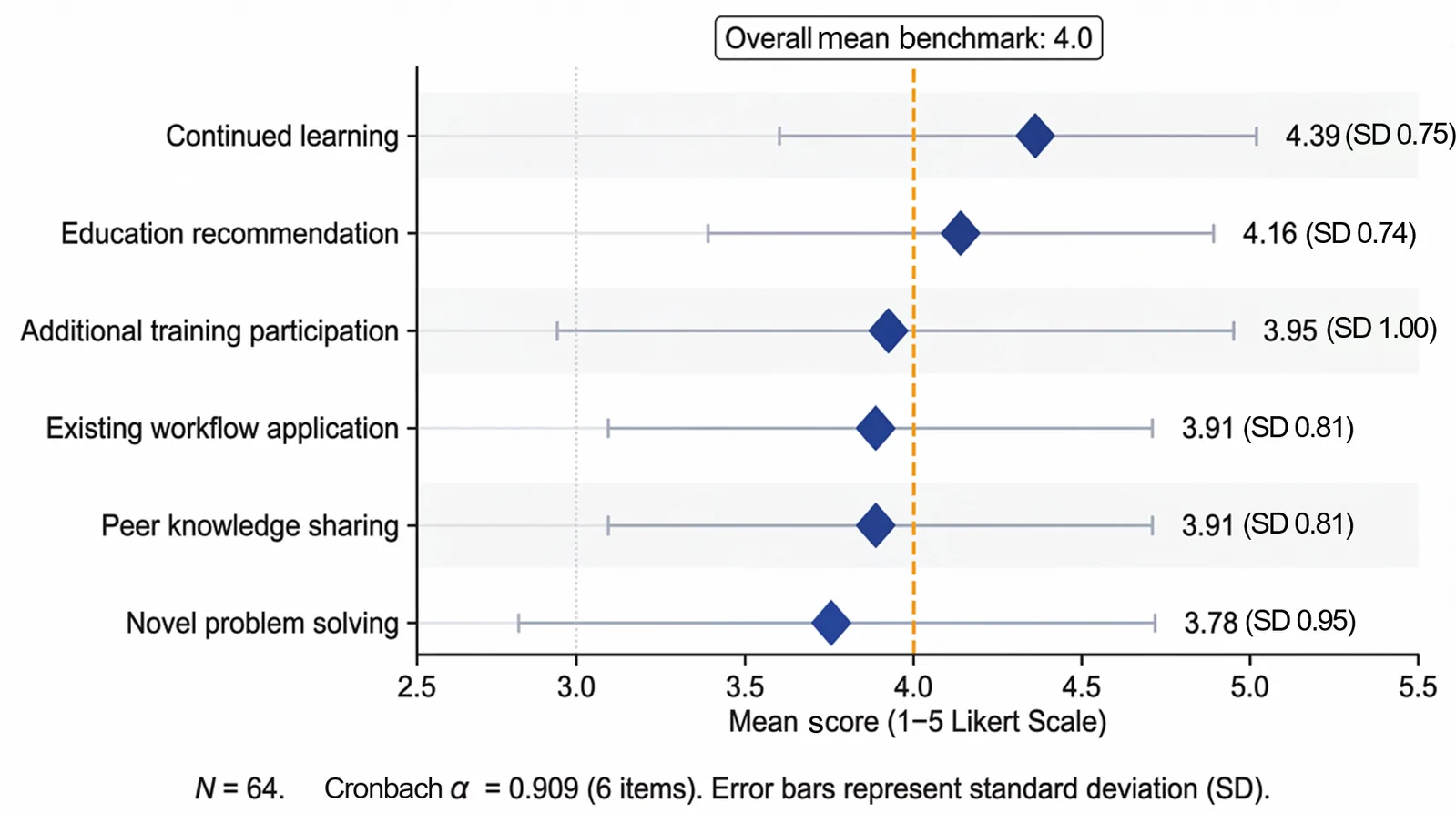
Transfer of learning intentions across six items measured on a 5-point Likert scale (N=64; Cronbach α=0.909, 6 items), with the dashed line indicating the overall mean benchmark of 4.0. Error bars represent SD.

Perceived changes (S10; α=0.928; Figure 4) showed high perceived knowledge improvement (mean 4.27, SD 0.62) and technology acceptance (mean 4.20, SD 0.62), but lower workplace application confidence (mean 3.50, SD 0.84), reinforcing the knowledge-practice gap observed in S6. Workplace application intention (S11; α=0.909; Figure 5) was high overall (mean 4.02, SD 0.70), with continued learning rated highest (mean 4.39, SD 0.75) and novel problem solving rated lowest (mean 3.78, SD 0.95).

### Participants’ Self-Reported Growth (Open-Ended Responses)

All 64 posttraining respondents answered the open-ended question: “What aspect of change or growth did you most strongly feel through this 8-week program?” Eight responses (12.5%) were too brief to classify; the remaining 56 responses were grouped by the research team into 5 recurring themes. Because 8 of these responses expressed 2 distinct themes (none was assigned to more than 2), this grouping yielded 64 theme-level assignments in total. The most prevalent theme was conceptual clarity (24 assignments, 37.5% of all 64 respondents), with participants reporting that previously abstract concepts, particularly MCP and RAG, became concrete and actionable. The second was confidence and reduced fear (14 assignments, 21.9%); participants described a shift from anxiety about AI to self-efficacy, with representative responses including “the vague fear I had has been resolved to some extent” and “my first goal was confidence recovery, and I fully achieved it.” Third, hands-on tool experience (12 assignments, 18.8%) was frequently cited, with participants specifically naming vibe coding, Cursor, and Claude Code as transformative experiences; notably, several nondevelopers described coding for the first time. Fourth, workplace application ideation (10 assignments, 15.6%) emerged, with participants reporting that they began identifying specific AI applications in their own work contexts. Finally, a smaller group reported a general attitude shift (4 assignments, 6.2%).

## Discussion

### Principal Findings

This study describes the design, implementation, and outcomes of an 8-week intensive generative AI training program for a multidisciplinary hospital workforce. To our knowledge, this is the first study to report both the design and the pre-post evaluation of a hospital-workforce program integrating 4 advanced, agent-level generative AI technologies, MCP, RAG, LangGraph orchestration, and AI agent design, across both IT and non-IT roles, spanning IT specialists, clinicians, HIM, researchers, and administrative staff. This advances AI education research beyond its prevailing focus on AI literacy and prompt engineering [10-13] toward agent-level competencies, and, through the capstone-to-deployment pathway, links classroom learning to institutional adoption. Five principal lessons emerged from the convergence of quantitative survey data and participants’ open-ended reflections.

### Lesson 1: Center the Curriculum on Agent-Level Technologies—The Unfamiliar Yields the Greatest Returns

The program’s most consequential design decision was allocating 37% of instructional time to MCP, a technology virtually none of the participants had encountered. The quantitative data validated this bet: MCP showed the largest between-group self-efficacy difference across all professional groups (*d*=1.57; Table 2), and the 3 novel domains (MCP, medical Q&A, and AI agent design) consistently outperformed foundational topics. The open-ended responses corroborated this pattern—MCP and RAG were the most frequently named technologies when participants described their growth, and the dominant theme was that “previously abstract concepts became concrete and actionable.” This inverse relationship between baseline familiarity and learning yield is consistent with self-efficacy theory’s prediction that mastery experiences are most impactful where prior competency is lowest [19], and suggests that training programs should resist the temptation to stay at the prompt-engineering level.

### Lesson 2: Include Non-IT Professionals—They Benefit Most and Bring Irreplaceable Domain Expertise

Non-IT professionals showed consistently larger between-group differences than IT specialists across most domains (Table S2 in Multimedia Appendix 1), particularly for MCP (clinicians: *d*=2.88; HIM: *d*=2.45). This finding challenges the assumption that advanced AI training should be restricted to technical staff. Qualitatively, nondevelopers described transformative experiences: several reported coding for the first time through vibe coding, and one described the capstone as an opportunity to “bring MCP and RAG into hospital work and implement visible results through hands-on practice—a fundamentally different experience from watching lecture slides.” The mixed-expertise team structure, which received the highest satisfaction rating (mean 3.98, SD 0.84), provided the technical scaffolding non-IT professionals needed while leveraging their domain knowledge for health care-specific applications.

### Lesson 3: Expect a Knowledge-Practice Gap—and Design for It

Despite significant self-efficacy gains (*r*=.574) and high perceived knowledge improvement (mean 4.27, SD 0.62), job-specific competency remained below the scale midpoint (2.77/5) and workplace application confidence was rated only 3.50—a gap that converged across 3 independent measures (S6, S10, and S11). Yet, the open-ended responses revealed a more nuanced picture: 15.6% of respondents spontaneously reported generating specific workplace application ideas, and participants described shifts from passive understanding to active problem identification (“I started thinking about improvements to the systems we currently operate”). The Unified Theory of Acceptance and Use of Technology (UTAUT) framework [22] suggests that training without corresponding organizational change management may produce motivated but unsupported learners [34,35]; health care settings compound this through restricted electronic medical record (EMR) environments, data governance requirements, and concerns about LLM hallucination. The gap is not a failure of training but a predictable transition challenge—one that infrastructure support (funded tool subscriptions, secure internal cloud environments) and structured posttraining mentoring can help bridge [36,37].

### Lesson 4: Plan for Differentiated Pacing Across Heterogeneous Cohorts

Curriculum pacing was rated lowest among satisfaction domains (mean 2.91/5.0, SD 1.11), even as overall satisfaction was substantially higher (4.03/5.0). This signals that participants valued the program but struggled to keep pace—a predictable tension when teaching rapidly evolving technologies to professionals ranging from experienced developers to clinical staff encountering code for the first time. The qualitative data illuminated both sides: some participants celebrated “coding fearlessly for the first time,” while the pacing score suggests others felt left behind. In practice, hands-on exercises were already split into application and development tracks matched to baseline skill; future iterations should additionally consider prerequisite assessment and supplementary catch-up sessions to accommodate varying baseline competencies.

Teaching a mixed-expertise cohort posed distinct challenges. IT and health-information staff were released from their regular duties to attend, whereas some clinicians and researchers used personal annual leave. At the outset, the wide range in baseline skill, from staff who had never coded to experienced IT specialists, was anticipated as a key challenge; 2 design choices mitigated it (Methods): the curriculum minimized AI theory in favor of practical concepts, agentic methods, and concrete MCP use cases, and each capstone team paired an intact IT-subdepartment core with non-IT domain members. Collaboration across backgrounds still required deliberate team-level scaffolding, and recruitment skewed toward IT staff, consistent with the program’s IT-first prioritization and department-based, voluntary enrollment—with comparatively fewer clinical participants.

### Lesson 5: Capstone Projects Bridge Instruction and Institutional Adoption

The capstone component, supported by 3‐6 hours of weekly mentoring per team from a dedicated instructor, culminated in functional prototypes from 11 of 12 teams. The qualitative data revealed why: participants described the capstone as where abstract knowledge “became real”—the transition from understanding concepts to building something that works. More importantly, this bridge extended beyond the classroom: one capstone project has since advanced into institutional pilot use (AI-Docs; see Results). This progression from capstone prototype into pilot clinical use provides emerging Kirkpatrick level 3 evidence that training-generated projects can move into institutional adoption, suggesting the capstone model can serve as a pathway for AI innovation that outlasts the training period itself.

This program was conceived as the deliberate first phase of a staged, institution-wide strategy: we began with staff positioned to enable broad adoption (IT, health-information, related-department clinicians, and researchers), expecting their agent-level competency to seed hospital-wide AI deployment—a trajectory already visible in the AI-Docs pilot. Subsequent phases are planned to extend structured training to frontline clinicians and other departments and to embed the curriculum as a recurring continuing professional development (CPD) track, a required or elective module, or a template for other hospitals through the national framework that funded it. So framed, this voluntary first cohort is a starting point, not an endpoint: audiences not yet aware of agent-level AI are reached through later, role-targeted phases rather than left behind.

### Comparison With Prior Work

The observed between-group difference in AI knowledge self-efficacy (*r*=.574) is larger than improvements typically reported in prior digital health education studies, which have largely relied on uncontrolled before-after designs and reported heterogeneous, generally smaller gains [38]. The larger effects likely reflect the intensive, hands-on design (71% of on-site time practical; 84% experiential including the capstone), consistent with experiential learning evidence [19,23]. Recent ChatGPT education studies for health care professionals report similar directions but with shorter interventions and smaller effects [11,12]; our previous health informatics analyst education program at the same institution [18] provides a direct comparator, and the present study extends that evidence to advanced generative AI technologies.

The absence of significant attitudinal change (S2; *P*=.76) aligns with ceiling effects in health care AI attitude studies [13,39], where voluntary enrollees already hold favorable views (4.29/5). Notably, however, the qualitative data captured attitudinal shifts that the Likert scale missed: 21.9% of respondents spontaneously described reduced fear or increased confidence as their most significant growth—suggesting that self-efficacy gains may be a more sensitive indicator of training impact than attitudinal scales in already AI-positive populations. Future studies should recruit participants with more heterogeneous baseline attitudes [40] and consider AI adoption readiness [21] as an alternative construct.

### Limitations

This study has several important limitations that should be considered when interpreting the findings.

#### Study Design Constraints

The independent samples design, necessitated by anonymous data collection, prevents individual-level tracking, precluding within-person effect sizes; future studies should consider self-generated identification codes to enable paired analysis [41]. The absence of a control group precludes causal attribution; improvements may partly reflect maturation or concurrent self-study. Additionally, the inverse relationship between baseline scores and improvement magnitude may partly reflect regression to the mean, particularly in this design where groups are not matched at the individual level. Subgroup comparisons by professional group also warrant caution beyond their exploratory, small-cell nature, because professional role was confounded with both baseline competency and career stage. The groups with the lowest baseline self-efficacy—HIM (pre mean 1.74, SD 0.44) and clinicians (1.92)—showed the largest gains, consistent with regression to the mean and ceiling effects, whereas researchers, despite being the most early-career group (none with ≥11 years of experience), reported the highest baseline and posttraining scores. Professional roles were, moreover, self-classified. The larger gains observed for non-IT groups therefore cannot be attributed to professional background independent of baseline ability and experience.

#### Attrition and Selection Bias

Of 83 enrollees, 19 (22.9%) did not respond to the posttraining survey, and these individuals may have differed systematically from respondents. A worst-case sensitivity analysis demonstrated that primary outcomes remain statistically significant even assuming zero training effect in all nonrespondents (Table S7 in Multimedia Appendix 1). Nevertheless, self-selection bias limits generalizability: voluntary enrollees at affiliated institutions with high baseline positive-attitude scores (4.29/5) may not represent the broader health care workforce. Importantly, posttraining nonresponse was not evenly distributed across professional groups: retention was lowest among IT specialists (33 of 48; 68.8%) and higher among non-IT groups (78% ‐100%). Because the subgroup analyses contrast non-IT groups against IT specialists, this differential attrition may modestly inflate the apparent non-IT advantage, and the comparable cross-sectional occupational distributions should be interpreted with caution given limited statistical power.

#### Measurement Limitations

All outcomes relied on self-report measures subject to social desirability bias [42]. Self-efficacy assessments administered immediately after training may be inflated by posttraining euphoria, and the Dunning-Kruger effect is particularly relevant for novel technology domains where participants may lack sufficient expertise to calibrate their self-assessment accurately. Future studies should complement self-efficacy measures with objective performance assessments [26]. The survey instrument was developed without formal content validity index (CVI) assessment or pilot testing, limiting construct validity evidence beyond the post hoc psychometric analyses reported in Tables S5-S6 in Multimedia Appendix 1; future iterations should establish CVI ≥.80 via a priori expert panel review.

#### Generalizability and Temporal Scope

Despite multisite delivery, participants were drawn primarily from a single tertiary academic medical center, limiting external validity. Short-term assessment does not capture whether gains persist; longitudinal follow-up at 3‐6 months is needed to assess durability and actual behavioral transfer [43].

### Conclusions

This study suggests that transforming a multidisciplinary hospital workforce into AI-capable professionals is achievable through intensive, hands-on training centered on agent-level technologies. The curriculum’s investment in MCP, the technology anticipated to underpin future health care AI infrastructure, was associated with the largest self-efficacy gains, and non-IT professionals showed the largest between-group differences, challenging the assumption that advanced AI training should be restricted to technical staff. The team-based capstone model proved particularly valuable, with prototypes that progressed toward institutional adoption, providing early evidence that training programs can serve as a pathway for institutional AI innovation. However, the knowledge-practice gap identified across multiple measures underscores that training alone is insufficient; posttraining support structures, mentoring, communities of practice, and supervised implementation are essential to translate self-efficacy gains into sustained workplace practice. Future research should use longitudinal designs with matched cohorts to assess the durability of training effects and to determine whether capstone-to-deployment pathways can be replicated across institutions.

## Supplementary material

10.2196/97822 Multimedia Appendix 1 Tables S1-S8 (S1: training curriculum by week; S2: S4 full domain statistics by subgroup with 95% CIs; S3: posttraining item-level results; S4: post-hoc power analysis; S5: exploratory factor analysis results; S6: item-total correlation analysis; S7: worst-case nonresponse sensitivity analysis; S8: capstone project descriptions and outcomes).

10.2196/97822 Multimedia Appendix 2 Detailed session-level training syllabus (per-session learning objectives, hands-on exercises, and tools).

10.2196/97822 Multimedia Appendix 3 Capstone project evaluation rubric (standardized 100-point rubric used by the assessment panel).

10.2196/97822 Multimedia Appendix 4 Open-ended survey responses (English translations of all 64 free-text responses to the posttraining self-perceived growth question).

10.2196/97822 Checklist 1 STROBE checklist for cross-sectional studies.

## Abbreviations

BEME: Best Evidence Medical Education
CPD: continuing professional development
CVI: content validity index
EFA: exploratory factor analysis
EMR: electronic medical record
HIM: health information manager
KOHI: Korea Human Resource Development Institute for Health & Welfare
LLM: large language model
MCP: model context protocol
Q&A: question and answer
RAG: retrieval-augmented generation
RQ: research question
STROBE: Strengthening the Reporting of Observational Studies in Epidemiology
UTAUT: Unified Theory of Acceptance and Use of Technology

## References

[1] Seo J, Choi D, Kim T (2024). Evaluation framework of large language models in medical documentation: development and usability study. J Med Internet Res.

[2] Topol EJ (2019). High-performance medicine: the convergence of human and artificial intelligence. Nat Med.

[3] Thirunavukarasu AJ, Ting DSJ, Elangovan K, Gutierrez L, Tan TF, Ting DSW (2023). Large language models in medicine. Nat Med.

[4] He J, Baxter SL, Xu J, Xu J, Zhou X, Zhang K (2019). The practical implementation of artificial intelligence technologies in medicine. Nat Med.

[5] Lewis P, Perez E, Piktus A, Petroni F, Karpukhin V, Goyal N (2020). Retrieval-augmented generation for knowledge-intensive NLP tasks. https://proceedings.neurips.cc/paper/2020/file/6b493230205f780e1bc26945df7481e5-Paper.pdf.

[6] (2024). What is the model context protocol (MCP)?. Model Context Protocol.

[7] (2024). LangGraph overview. LangChain Docs.

[8] Sallam M (2023). ChatGPT utility in healthcare education, research, and practice: systematic review on the promising perspectives and valid concerns. Healthcare (Basel).

[9] Laupichler MC, Aster A, Schirch J, Raupach T (2022). Artificial intelligence literacy in higher and adult education: a scoping literature review. Comput Educ Artif Intell.

[10] Gordon M, Daniel M, Ajiboye A (2024). A scoping review of artificial intelligence in medical education: BEME Guide No. 84. Med Teach.

[11] Wu Y, Zheng Y, Feng B, Yang Y, Kang K, Zhao A (2024). Embracing ChatGPT for medical education: exploring its impact on doctors and medical students. JMIR Med Educ.

[12] Hu JM, Liu FC, Chu CM, Chang YT (2023). Health care trainees’ and professionals’ perceptions of ChatGPT in improving medical knowledge training: rapid survey study. J Med Internet Res.

[13] Tangadulrat P, Sono S, Tangtrakulwanich B (2023). Using ChatGPT for clinical practice and medical education: cross-sectional survey of medical students’ and physicians’ perceptions. JMIR Med Educ.

[14] Sapci AH, Sapci HA (2020). Artificial intelligence education and tools for medical and health informatics students: systematic review. JMIR Med Educ.

[15] Preiksaitis C, Rose C (2023). Opportunities, challenges, and future directions of generative artificial intelligence in medical education: scoping review. JMIR Med Educ.

[16] Charow R, Jeyakumar T, Younus S (2021). Artificial intelligence education programs for health care professionals: scoping review. JMIR Med Educ.

[17] Lee YM, Kim S, Lee YH (2024). Defining medical AI competencies for medical school graduates: outcomes of a delphi survey and medical student/educator questionnaire of South Korean medical schools. Acad Med.

[18] Lee KH, Lee JH, Lee Y (2024). Impact of health informatics analyst education on job role, career transition, and skill development: survey study. JMIR Med Educ.

[19] Bandura A (1997). Self-Efficacy: The Exercise of Control.

[20] Compeau DR, Higgins CA (1995). Computer self-efficacy: development of a measure and initial test. MIS Q.

[21] Davis FD (1989). Perceived usefulness, perceived ease of use, and user acceptance of information technology. MIS Q.

[22] Venkatesh V, Morris MG, Davis GB, Davis FD (2003). User acceptance of information technology: toward a unified view1. MIS Q.

[23] Kolb DA (2015). Experiential Learning: Experience as the Source of Learning and Development.

[24] Kirkpatrick DL (1977). Evaluating training programs: evidence vs. proof. Train Dev J.

[25] Kirkpatrick JD, Kirkpatrick WK (2016). Kirkpatrick’s Four Levels of Training Evaluation.

[26] Yardley S, Dornan T (2012). Kirkpatrick’s levels and education “evidence”. Med Educ.

[27] Alhassan AI (2022). Implementing faculty development programs in medical education utilizing Kirkpatrick’s model. Adv Med Educ Pract.

[28] von Elm E, Altman DG, Egger M (2007). The Strengthening the Reporting of Observational Studies in Epidemiology (STROBE) statement: guidelines for reporting observational studies. Lancet.

[29] Wang L, Ma C, Feng X (2024). A survey on large language model based autonomous agents. Front Comput Sci.

[30] Hollander M, Wolfe DA, Chicken E (2014). Nonparametric Statistical Methods.

[31] Rosenthal R (1991). Meta-Analytic Procedures for Social Research.

[32] Cohen J (1988). Statistical Power Analysis for the Behavioral Sciences.

[33] Nunnally JC, Bernstein IH (1994). Psychometric Theory.

[34] Hassan M, Kushniruk A, Borycki E (2024). Barriers to and facilitators of artificial intelligence adoption in health care: scoping review. JMIR Hum Factors.

[35] Greenhalgh T, Wherton J, Papoutsi C (2017). Beyond adoption: a new framework for theorizing and evaluating nonadoption, abandonment, and challenges to the scale-up, spread, and sustainability of health and care technologies. J Med Internet Res.

[36] Pfeffer J, Sutton RI (2000). The Knowing-Doing Gap: How Smart Companies Turn Knowledge into Action.

[37] Grimshaw JM, Eccles MP, Lavis JN, Hill SJ, Squires JE (2012). Knowledge translation of research findings. Implement Sci.

[38] Tudor Car L, Kyaw BM, Nannan Panday RS (2021). Digital health training programs for medical students: scoping review. JMIR Med Educ.

[39] Chen M, Zhang B, Cai Z (2022). Acceptance of clinical artificial intelligence among physicians and medical students: a systematic review with cross-sectional survey. Front Med (Lausanne).

[40] Wartman SA, Combs CD (2019). Reimagining medical education in the age of AI. AMA J Ethics.

[41] Yurek LA, Vasey J, Sullivan Havens D (2008). The use of self-generated identification codes in longitudinal research. Eval Rev.

[42] Podsakoff PM, MacKenzie SB, Lee JY, Podsakoff NP (2003). Common method biases in behavioral research: a critical review of the literature and recommended remedies. J Appl Psychol.

[43] Salas E, Tannenbaum SI, Kraiger K, Smith-Jentsch KA (2012). The science of training and development in organizations: what matters in practice. Psychol Sci Public Interest.

