# Supplementary material for: Teaching Model Context Protocol, Retrieval-Augmented Generation, and AI Agents to a Multidisciplinary Hospital Workforce: Single-Group Pre-Post Survey Study
Source: JMIR Med Educ. 2026 Sep 11;12:e97822. doi: 10.2196/97822 (PMC13568689; doi:10.2196/97822)
Supplement: Supplementary file 1 [file mededu-v12-e97822-s001.docx]

## Table S1. Training Curriculum by Week

| Week | Date | Session | Duration (h) | Topic | Format |
| --- | --- | --- | --- | --- | --- |
| 1 | Sep 8 (Mon) | AM | 3 | Foundation models overview: AI history, deep learning, foundation model architectures | Lecture |
| 1 | Sep 8 (Mon) | PM | 4 | Prompt engineering and development environment setup: healthcare domain-specific prompting, Cursor IDE installation | Hands-on |
| 2 | Sep 18 (Thu) | AM | 3 | Medical document Q&A system: PDF chunking, RAG implementation, vibe coding with Cursor | Hands-on |
| 2 | Sep 18 (Thu) | PM | 4 | LangGraph workflow design: graph-based design patterns, Ollama integration, clinical process modeling | Hands-on |
| 3 | Sep 22 (Mon) | AM | 3 | MCP introduction and healthcare applications: MCP architecture (Tools/Resources/Prompts), medical use cases | Lecture + Hands-on |
| 3 | Sep 22 (Mon) | PM | 4 | Vector databases and RAG agents: LLM concepts, RAG agent implementation, LangGraph agent development | Hands-on |
| 4 | Oct 13 (Mon) | AM | 3 | AI agent architecture and design principles: agent types for clinical workflows, safety and explainability, architecture diagramming, capstone scenario selection | Lecture + Hands-on |
| 4 | Oct 13 (Mon) | PM | 4 | MCP server development and team strategy: LangChain/LangGraph frameworks, AI agent architecture, capstone planning | Hands-on |
| 5–7 | Oct 20–Nov 7 | Self-directed | 25^a^ | Team-based capstone projects I–III: MVP development → refinement → quality evaluation | Project |
| 8 | Nov 11 (Tue) | AM | 3 | MCP host development and workflow integration: medical workflow orchestration, advanced LangGraph patterns | Hands-on |
| 8 | Nov 11 (Tue) | PM-1 | 1 | Institutional AI services overview: current and future AI applications at Asan Medical Center | Lecture |
| 8 | Nov 11 (Tue) | PM-2 | 3 | Capstone project presentations and awards ceremony (12 teams) | Presentation |

**Total: 56 hours** (on-site: 31 hours^b^ + team project: 25 hours)

^a^ Team project hours estimated based on 3-week duration with approximately 8 hours/week of self-directed work.

^b On-site instructional hours (31 h) comprise the Week 1–4 sessions (28 h) and the Week 8 morning session (3 h); the Week 8 institutional AI overview (1 h) and the capstone presentation and awards ceremony (3 h) are listed for completeness and are not counted as instructional time.^

**Technology-specific instructional hours (on-site, 31 hours):**

| Technology | Hours | % of on-site time | Key sessions |
| --- | --- | --- | --- |
| MCP | 11.5 | 37% | Weeks 3 (AM), 4 (PM), 8 (AM) |
| LangGraph workflow | 8.5 | 27% | Weeks 2 (PM), 4 (PM), 8 (AM) |
| Foundation models & prompt engineering | 7.0 | 23% | Week 1 (AM+PM) |
| RAG | 5.0 | 16% | Weeks 2 (AM), 3 (PM) |
| Medical Q&A systems | 5.0 | 16% | Weeks 2 (AM), 4 (AM) |
| AI agent design | 4.0 | 13% | Weeks 4 (AM, PM) |

Note: Some sessions covered multiple technologies; percentages exceed 100% due to overlap.

Instructional format: Lecture 9 hours (29%) vs Hands-on practice 22 hours (71%).

## Table S2. S4 AI Knowledge Self-Efficacy by Domain and Professional Group: Full Statistics

*Eight domains of the S4 AI Knowledge Self-Efficacy scale (S4-1: Basic AI understanding, S4-2: Prompt engineering, S4-3: RAG, S4-4: Local LLM deployment, S4-5: LangGraph workflow, S4-6: MCP, S4-7: Medical Q&A systems, S4-8: AI agent design). Pre: n = 83; Post: n = 64.*

| Domain | Group | Pre n | Post n | Pre M (SD) | Post M (SD) | Δ | Cohen’s d | 95% CI | U | p |
| --- | --- | --- | --- | --- | --- | --- | --- | --- | --- | --- |
| **Basic AI understanding** | IT specialists | 48 | 33 | 3.15 (0.90) | 3.67 (0.60) | +0.52 | 0.66 | [0.20, 1.11] | 546.5 | .010 |
|  | HIM | 10 | 9 | 2.50 (1.08) | 2.89 (0.78) | +0.39 | 0.41 | [−0.50, 1.32] | 35.5 | .441 |
|  | Clinicians | 9 | 7 | 2.33 (1.41) | 3.29 (1.11) | +0.95 | 0.74 | [−0.29, 1.76] | 19.0 | .191 |
|  | Researchers | 9 | 8 | 4.00 (0.87) | 4.25 (0.71) | +0.25 | 0.31 | [−0.64, 1.27] | 30.0 | .572 |
|  | Admin | 7 | 7 | 3.00 (0.82) | 3.57 (0.79) | +0.57 | 0.71 | [−0.37, 1.80] | 16.0 | .268 |
| **Prompt engineering** | IT specialists | 48 | 33 | 2.81 (0.96) | 3.58 (0.75) | +0.76 | 0.87 | [0.40, 1.33] | 448.0 | <.001 |
|  | HIM | 10 | 9 | 1.90 (0.88) | 3.33 (1.00) | +1.43 | 1.53 | [0.49, 2.57] | 13.5 | .009 |
|  | Clinicians | 9 | 7 | 2.00 (1.22) | 3.43 (0.98) | +1.43 | 1.27 | [0.18, 2.36] | 12.5 | .040 |
|  | Researchers | 9 | 8 | 3.44 (0.88) | 4.12 (0.64) | +0.68 | 0.87 | [−0.13, 1.88] | 19.5 | .098 |
|  | Admin | 7 | 7 | 3.14 (0.69) | 3.57 (0.98) | +0.43 | 0.51 | [−0.56, 1.57] | 17.5 | .375 |
| **RAG** | IT specialists | 48 | 33 | 2.52 (1.05) | 3.61 (0.75) | +1.09 | 1.15 | [0.68, 1.63] | 354.0 | <.001 |
|  | HIM | 10 | 9 | 1.70 (0.67) | 3.00 (1.22) | +1.30 | 1.34 | [0.33, 2.34] | 16.5 | .017 |
|  | Clinicians | 9 | 7 | 2.11 (1.36) | 3.43 (1.27) | +1.32 | 0.99 | [−0.06, 2.05] | 15.0 | .082 |
|  | Researchers | 9 | 8 | 3.11 (1.27) | 4.12 (0.64) | +1.01 | 0.99 | [−0.03, 2.01] | 18.5 | .089 |
|  | Admin | 7 | 7 | 2.71 (1.11) | 3.43 (0.79) | +0.71 | 0.74 | [−0.35, 1.83] | 15.0 | .224 |
| **Local LLM deployment** | IT specialists | 48 | 33 | 3.17 (1.23) | 3.97 (0.81) | +0.80 | 0.75 | [0.29, 1.20] | 499.5 | .003 |
|  | HIM | 10 | 9 | 2.50 (1.43) | 3.44 (0.88) | +0.94 | 0.78 | [−0.16, 1.72] | 26.0 | .122 |
|  | Clinicians | 9 | 7 | 2.22 (1.48) | 3.71 (0.76) | +1.49 | 1.22 | [0.13, 2.30] | 15.0 | .075 |
|  | Researchers | 9 | 8 | 3.78 (1.30) | 4.38 (0.74) | +0.60 | 0.55 | [−0.42, 1.53] | 27.5 | .412 |
|  | Admin | 7 | 7 | 2.71 (1.11) | 3.71 (0.76) | +1.00 | 1.05 | [−0.08, 2.18] | 12.0 | .108 |
| **LangGraph workflow** | IT specialists | 48 | 33 | 2.10 (0.99) | 3.06 (1.00) | +0.96 | 0.96 | [0.49, 1.43] | 407.0 | <.001 |
|  | HIM | 10 | 9 | 1.50 (0.85) | 2.67 (0.87) | +1.17 | 1.36 | [0.35, 2.37] | 14.5 | .010 |
|  | Clinicians | 9 | 7 | 1.67 (1.12) | 2.57 (0.79) | +0.90 | 0.91 | [−0.13, 1.96] | 13.5 | .052 |
|  | Researchers | 9 | 8 | 2.33 (1.32) | 3.62 (1.06) | +1.29 | 1.07 | [0.04, 2.10] | 15.5 | .047 |
|  | Admin | 7 | 7 | 2.14 (1.07) | 3.29 (0.76) | +1.14 | 1.23 | [0.08, 2.39] | 10.0 | .054 |
| **MCP** | IT specialists | 48 | 33 | 2.08 (1.01) | 3.33 (0.78) | +1.25 | 1.36 | [0.87, 1.85] | 289.0 | <.001 |
|  | HIM | 10 | 9 | 1.10 (0.32) | 2.44 (0.73) | +1.34 | 2.45 | [1.23, 3.67] | 3.0 | <.001 |
|  | Clinicians | 9 | 7 | 1.56 (0.73) | 3.43 (0.53) | +1.87 | 2.88 | [1.42, 4.33] | 2.0 | .002 |
|  | Researchers | 9 | 8 | 1.89 (1.05) | 3.88 (0.83) | +1.99 | 2.07 | [0.87, 3.28] | 6.0 | .004 |
|  | Admin | 7 | 7 | 1.71 (0.95) | 3.29 (0.76) | +1.57 | 1.83 | [0.55, 3.11] | 5.5 | .014 |
| **Medical Q&A systems** | IT specialists | 48 | 33 | 1.94 (0.89) | 3.39 (0.86) | +1.46 | 1.66 | [1.15, 2.18] | 217.0 | <.001 |
|  | HIM | 10 | 9 | 1.70 (0.95) | 2.44 (1.01) | +0.74 | 0.76 | [−0.18, 1.70] | 27.0 | .126 |
|  | Clinicians | 9 | 7 | 1.89 (1.17) | 3.14 (0.69) | +1.25 | 1.27 | [0.17, 2.36] | 12.5 | .040 |
|  | Researchers | 9 | 8 | 2.67 (1.50) | 4.00 (0.76) | +1.33 | 1.10 | [0.07, 2.13] | 17.0 | .066 |
|  | Admin | 7 | 7 | 1.71 (0.95) | 3.43 (0.53) | +1.71 | 2.22 | [0.85, 3.60] | 4.0 | .007 |
| **AI agent design** | IT specialists | 48 | 33 | 1.73 (0.89) | 3.03 (0.88) | +1.30 | 1.46 | [0.96, 1.96] | 259.0 | <.001 |
|  | HIM | 10 | 9 | 1.00 (0.00) | 2.11 (0.78) | +1.11 | 2.07 | [0.93, 3.21] | 10.0 | .001 |
|  | Clinicians | 9 | 7 | 1.56 (0.88) | 2.71 (0.76) | +1.16 | 1.40 | [0.28, 2.51] | 10.5 | .023 |
|  | Researchers | 9 | 8 | 2.22 (1.48) | 3.75 (0.71) | +1.53 | 1.29 | [0.23, 2.35] | 14.0 | .034 |
|  | Admin | 7 | 7 | 1.43 (0.79) | 2.86 (0.69) | +1.43 | 1.93 | [0.63, 3.23] | 5.0 | .011 |

HIM = health information managers; Admin = administrative staff; Δ = post − pre mean difference. Cohen’s d computed using pooled standard deviation; 95% CI derived from Hedges’ standard error formula (SE = √[(n₁+n₂)/(n₁n₂) + d²/(2(n₁+n₂−2))]).

Bold indicates domain names. U and p values from Mann-Whitney U tests are provided for reference; however, given the exploratory nature of these subgroup analyses and the absence of multiple-comparisons correction, these should not be interpreted as confirmatory. Cohen’s d and 95% CIs are the primary descriptive statistics (see Table 3 in main text).

## Table S3. Post-Training Outcomes: Item-Level Results (N = 64)

### Panel A. Training Satisfaction (S9; Cronbach’s α = .939)

| Domain (α) | Item | M | SD |
| --- | --- | --- | --- |
| **Curriculum Design** (α = .849) |  | 2.95 | 0.87 |
|  | Curriculum structure appropriateness | — | — |
|  | Content difficulty level | — | — |
|  | Learning pace appropriateness | 2.91 | 1.11 |
| **Content & Practice** (α = .802) |  | 3.40 | 0.67 |
| **Instructor Quality** (α = .862) |  | 3.91 | 0.72 |
| **Teaching Methods** (α = .820) |  | 3.58 | 0.69 |
| **Team Project** (α = .789) |  | 3.98 | 0.84 |
|  | Collaborative experience | 4.05 | — |
| **Learning Environment** (α = .912) |  | 3.07 | 1.06 |
| **Overall Satisfaction** (single item) | Overall program satisfaction | 4.03 | 0.85 |

Note: Domain means are averages across constituent items. Individual item statistics shown where cited in the main text; full item-level data available from the corresponding author.

### Panel B. Perceived Changes (S10; Cronbach’s α = .928)

| Construct | Item | M | SD |
| --- | --- | --- | --- |
| **Knowledge** | Knowledge improvement (이해도향상) | 4.27 | 0.62 |
|  | AI terminology and concepts (AI용어구체화) | 4.00 | 0.69 |
|  | AI technology understanding (AI기술이해) | 3.75 | 0.69 |
| **Skills** | Skill enhancement (활용능력향상) | 4.05 | 0.72 |
| **Attitudes** | Technology acceptance (수용의지강화) | 4.20 | 0.62 |
|  | Proactive attitude toward AI (적극적태도) | 3.91 | 0.66 |
|  | Reduced AI-related anxiety (불안감감소) | 3.89 | 0.76 |
|  | Reduced replacement concern (불안감감소2) | 3.86 | 0.66 |
| **Application** | Confidence in attempting AI tasks (업무시도자신감) | 3.62 | 0.83 |
|  | Workplace application confidence (업무적용방법) | 3.50 | 0.84 |
|  | **Scale total** | **3.90** | **0.56** |

### Panel C. Workplace Application Intention (S11; Cronbach’s α = .909)

| Item | M | SD |
| --- | --- | --- |
| Continued learning intention (지속학습) | 4.39 | 0.75 |
| Education recommendation to peers (교육추천) | 4.16 | 0.74 |
| Additional training participation (추가교육참여) | 3.95 | 1.00 |
| Existing workflow application (기존업무활용) | 3.91 | 0.81 |
| Peer knowledge sharing (동료공유) | 3.91 | 0.81 |
| Novel problem-solving application (새문제해결) | 3.78 | 0.95 |
| **Scale total** | **4.02** | **0.70** |

Panel B items grouped by construct (Knowledge, Skills, Attitudes, Application); Panel C items sorted by descending mean.

## Table S4. Post-Hoc Power Analysis

| Section | Measure | Observed r | Post-hoc power | Interpretation |
| --- | --- | --- | --- | --- |
| S4 | AI Knowledge Self-Efficacy | .574 | >.999 | Adequately powered |
| S6 | Job-Specific AI Competency | .382 | .997 | Adequately powered |
| S5 | Digital Competency | .138 | .316 | Underpowered |
| S3 | AI Daily Usage | .122 | .250 | Underpowered |
| S2 | AI Attitudes | .025 | .060 | Severely underpowered |

Parameters: n₁ = 83, n₂ = 64, α = .05 (two-tailed), Mann-Whitney U test.

Minimum detectable effect size at 80% power: r = .24 (medium effect).

Note: This was a convenience sample; no a priori sample size calculation was performed. Power analysis is presented to contextualize nonsignificant findings. The study was adequately powered to detect large effects (r ≥ 0.5) but underpowered for small-to-medium effects (r < 0.24), suggesting that nonsignificant findings for S2, S3, and S5 should be interpreted cautiously.

## Table S5. Exploratory Factor Analysis Results

### Panel A. S4 AI Knowledge Self-Efficacy (N = 147, pooled pre+post)

**Adequacy**: KMO = .909; Bartlett’s test of sphericity: χ²(28) = 985.1, *p* < .001

**Factor extraction**: Kaiser criterion (eigenvalue > 1) extracted 1 factor. Eigenvalues: F1 = 5.546; F2 = 0.896 (< 1, not retained).

**Variance explained**: 65.1% (1 factor)

| Item | Factor Loading | Communality (h²) |
| --- | --- | --- |
| Basic AI understanding | .74 | .55 |
| Prompt engineering | .79 | .62 |
| RAG | .89 | .79 |
| Local LLM deployment | .75 | .56 |
| LangGraph workflow | .77 | .59 |
| MCP | .86 | .74 |
| Medical Q&A systems | .82 | .68 |
| AI agent design | .83 | .68 |

Extraction method: Principal Axis Factoring; rotation: direct oblimin. All items load in the expected positive direction (loadings presented as absolute values due to sign indeterminacy in factor analysis).

### Panel B. S6 Job-Specific AI Competency (N = 147, pooled pre+post)

**Adequacy**: KMO = .923; Bartlett’s test: χ²(21) = 1342.3, *p* < .001

**Factor extraction**: Kaiser criterion extracted 1 factor. Eigenvalues: F1 = 5.981; F2 = 0.392 (< 1).

**Variance explained**: 83.1% (1 factor)

| Item | Factor Loading | Communality (h²) |
| --- | --- | --- |
| Efficient AI tool use | .84 | .71 |
| AI solution implementation | .89 | .79 |
| Seeking technical support | .95 | .90 |
| AI workflow design | .91 | .82 |
| AI security management | .96 | .92 |
| AI development/testing | .89 | .78 |
| Active AI integration | .95 | .90 |

Note: Items are job-role-specific (7 items per professional group); pooled analysis uses all 5 professional groups (N = 83 pre + 64 post = 147 observations). Each professional group responded to a different set of 7 items tailored to their role; pooled statistics reflect aggregation across all groups.

### Panel C. S9 Training Satisfaction (N = 64, post-training only)

**Adequacy**: KMO = .854; Bartlett’s test: χ²(153) = 885.2, *p* < .001

**Factor extraction**: Kaiser criterion extracted 4 factors. Eigenvalues: F1 = 9.16, F2 = 2.05, F3 = 1.13, F4 = 1.03.

**Variance explained**: 53.6% total (F1 = 18.2%, F2 = 15.2%, F3 = 11.9%, F4 = 8.3%)

Note: N:p ratio = 64:18 = 3.6:1; results are treated as exploratory given suboptimal ratio (recommended ≥ 5:1). Factor structure is presented for descriptive purposes only.

| Factor | Label | Primary Items |
| --- | --- | --- |
| F1 | Instructor Quality | Instructor expertise, delivery, Q&A responsiveness, live demonstrations, interactive engagement |
| F2 | Learning Environment & Team | Team project experience, peer collaboration, platform stability, tool preparation |
| F3 | Hands-on Practice | Sufficient practice time, support availability during exercises |
| F4 | Curriculum Design | Program duration adequacy, difficulty progression, pacing |

### Panel D. S2 AI Attitudes — Two-Factor Structure (N = 147, pooled)

**Adequacy**: KMO = .768; Bartlett’s test: χ²(36) = 607.8, *p* < .001

**Factor extraction**: Kaiser criterion extracted 2 factors. Eigenvalues: F1 = 3.618, F2 = 2.064 (both > 1); single-factor model explains only 35.6% of variance with near-zero loadings for Q1–Q2.

**Interpretation**: Two-factor structure distinguishes *AI Apprehension* (Q1–Q3) from *Adaptation Confidence* (Q4–Q9), accounting for the low overall pre-training reliability (α = .657).

| Item | F1: Adaptation Confidence | F2: AI Apprehension |
| --- | --- | --- |
| Q1 (Replacement concern) | .13 | .62 |
| Q2 (Decision-making worry) | .10 | .49 |
| Q3 (Understanding necessity) | −.03 | .88 |
| Q4 (Adaptation readiness) | .81 | .01 |
| Q5 (Technology acceptance) | .76 | .06 |
| Q6 (Confidence in adoption) | .85 | .06 |
| Q7 (Proactive engagement) | .73 | −.11 |
| Q8 (Reduced anxiety) | .77 | −.03 |
| Q9 (Integration confidence) | .76 | .11 |

Extraction method: Principal Axis Factoring; rotation: direct oblimin. The 1-factor solution shows near-zero loadings for Q1 (.027) and Q2 (.016), indicating poor fit for these items in a unidimensional model.

## Table S6. Item-Total Correlation Analysis

### Panel A. S4 AI Knowledge Self-Efficacy

| Timepoint | α | Item | r (corrected) | α if deleted |
| --- | --- | --- | --- | --- |
| Pre (N=83) | **.909** | Basic AI understanding | .71 | .897 |
|  |  | Prompt engineering | .66 | .901 |
|  |  | RAG | .84 | .885 |
|  |  | Local LLM deployment | .70 | .901 |
|  |  | LangGraph workflow | .71 | .897 |
|  |  | MCP | .73 | .895 |
|  |  | Medical Q&A systems | .64 | .902 |
|  |  | AI agent design | .68 | .900 |
| Post (N=64) | **.915** | Basic AI understanding | .75 | .902 |
|  |  | Prompt engineering | .76 | .901 |
|  |  | RAG | .74 | .902 |
|  |  | Local LLM deployment | .64 | .910 |
|  |  | LangGraph workflow | .58 | .917 |
|  |  | MCP | .78 | .900 |
|  |  | Medical Q&A systems | .81 | .896 |
|  |  | AI agent design | .74 | .902 |

All corrected item-total correlations ≥ .58, exceeding the recommended threshold of .30.

### Panel B. S2 AI Attitudes (raw scores, pre-reverse coding)

| Timepoint | α | Item | r (corrected) | α if deleted |
| --- | --- | --- | --- | --- |
| Pre (N=83) | **.657** | Q1 (Replacement concern) | .21 | .663 |
|  |  | Q2 (Decision-making worry) | .14^† | .694 |
|  |  | Q3 (Understanding necessity) | .33 | .632 |
|  |  | Q4 (Adaptation readiness) | .44 | .612 |
|  |  | Q5 (Technology acceptance) | .31 | .635 |
|  |  | Q6 (Confidence in adoption) | .40 | .620 |
|  |  | Q7 (Proactive engagement) | .52 | .597 |
|  |  | Q8 (Reduced anxiety) | .52 | .599 |
|  |  | Q9 (Integration confidence) | .40 | .614 |
| Post (N=64) | **.782** | Q1 | .28 | .794 |
|  |  | Q2 | .29 | .791 |
|  |  | Q3 | .56 | .748 |
|  |  | Q4 | .57 | .749 |
|  |  | Q5 | .56 | .751 |
|  |  | Q6 | .58 | .749 |
|  |  | Q7 | .55 | .753 |
|  |  | Q8 | .49 | .760 |
|  |  | Q9 | .51 | .756 |

^† Q2 r = .14, *p* = .199 (non-significant). All other items significant at *p* < .05. Pre-training Q1 and Q2 fall below the .30 threshold, consistent with the two-factor structure identified in Table S5, Panel D.

**S2 Subscale Reliability**: Apprehension subscale (Q1–Q3): α = .657 pre, .760 post. Confidence subscale (Q4–Q9): α = .828 pre, .865 post.

### Panel C. S5 Digital Competency

| Timepoint | α | Item | r (corrected) | α if deleted |
| --- | --- | --- | --- | --- |
| Pre (N=83) | **.831** | Computer proficiency | .66 | .805 |
|  |  | Cloud service experience | .55 | .833 |
|  |  | API/web service understanding | .78 | .729 |
|  |  | Data format literacy | .74 | .752 |
| Post (N=64) | **.820** | Computer proficiency | .56 | .814 |
|  |  | Cloud service experience | .58 | .805 |
|  |  | API/web service understanding | .79 | .705 |
|  |  | Data format literacy | .70 | .752 |

All corrected item-total correlations ≥ .55 (pre) and ≥ .56 (post).

### Panel D. S6 Job-Specific AI Competency (pooled across professional groups)

Recomputed using the verified sample (Pre N = 83, Post N = 64; 0 missing values)

| Timepoint | Pooled α | Item | r (corrected) | α if deleted |
| --- | --- | --- | --- | --- |
| Pre (N=83)83 | **.968** | Efficient AI tool use | .83 | .968 |
|  |  | AI solution implementation | .87 | .964 |
|  |  | Seeking technical support | .93 | .959 |
|  |  | AI workflow design | .87 | .964 |
|  |  | AI security management | .95 | .958 |
|  |  | AI development/testing | .84 | .966 |
|  |  | Active AI integration | .9392 | .960 |
| Post (N=64)64 | **.963962** | Efficient AI tool use | .7980 | .963961 |
|  |  | AI solution implementation | .8584 | .959958 |
|  |  | Seeking technical support | .9089 | .955954 |
|  |  | AI workflow design | .88 | .957955 |
|  |  | AI security management | .8988 | .956955 |
|  |  | AI development/testing | .8786 | .957 |
|  |  | Active AI integration | .9191 | .954953 |

Pooled analysis combines all five professional groups (IT specialists, Health Information Managers, Medical professionals, Researchers, and Other/administrative staff). Each respondent contributed seven job-specific items that share an identical 7-construct structure (item wording differs by professional group, but the underlying competency targeted by each item position is the same). No missing data were observed; pooling was performed at the respondent level. All corrected item-total correlations ≥ .80.

## Table S7. Sensitivity Analysis: Worst-Case Non-Response Imputation

**Rationale**: Of 83 pre-training participants, 19 did not respond to the post-training survey (response rate: 77.1%). To assess the robustness of primary findings to potential non-response bias, a worst-case sensitivity analysis was conducted: the 19 non-respondents were assigned post-training scores equal to their professional group’s pre-training mean, assuming no training benefit. The extended post-training group thus comprised 64 + 19 = 83 participants.

| Outcome | Scenario | N (pre/post) | U | Z | *p* | r | d |
| --- | --- | --- | --- | --- | --- | --- | --- |
| **S4 AI Knowledge** | Original | 83/64 | 875.5 | 6.956 | 3.50 × 10⁻¹² | .574 | 1.37 |
| **Self-Efficacy** | Worst-case | 83/83 | 1,654 | 5.789 | < .001 | .449 | 1.03 |
| **S6 Job-Specific** | Original | 83/64 | 1,470 | 4.634 | 3.59 × 10⁻⁶ | .382 | 0.85 |
| **AI Competency** | Worst-case | 83/83 | 1,978 | 3.996 | 6.5 × 10⁻⁵ | .318 | 0.72 |

**Interpretation**: Both primary outcomes remain statistically significant under the worst-case assumption, with large effect sizes maintained (S4: d = 1.03; S6: d = 0.72). This provides evidence that the main findings are robust to plausible non-response scenarios.

Non-respondents were distributed across professional groups proportional to the pre-training sample composition. Each non-respondent’s imputed post-training score equaled the observed pre-training mean for their professional group, representing the most conservative bound on training effectiveness.

## Table S8. Capstone Project Descriptions and Outcomes

*All 12 teams developed functional MCP-based AI prototypes during the 3-week capstone period (Weeks 5–7). Teams comprised mixed professional backgrounds (IT specialists, clinicians, health information managers, researchers, and administrative staff).*

| # | Project Title | Description | Core Technologies | Post-Training Status |
| --- | --- | --- | --- | --- |
| 1 | AI-Assisted Inpatient Diagnosis Classification Coding | Automated diagnostic code assignment for inpatient records using AI | MCP, RAG | Prototype |
| 2 | Medical Dictionary-Based RAG/Query System (“WorksBot”) | Chatbot for querying institutional medical terminology and knowledge bases | MCP, RAG, Vector DB | **Minister of Health and Welfare Award**; prototype — not yet in operational service (refinement planned) |
| 3 | Hospital Document Knowledge Retrieval RAG System | RAG-based search system for institutional documents and guidelines | MCP, RAG, Vector DB | Developed into institutional service (AI-Docs); in active pilot use (incl. Dept. of Nursing & Big Data Research Center); hospital-wide deployment planned 2026 |
| 4 | Patient Medication Information Chatbot | Patient-facing chatbot providing medication information using external drug databases | MCP, RAG | Prototype |
| 5 | Natural Language Clinical Data Retrieval System | Natural language interface for querying structured clinical databases | MCP, LangGraph | Prototype |
| 6 | Multimodal AI Dynamic Survey and Patient Support Platform | Patient-centered platform using multimodal AI for adaptive clinical surveys | MCP, LangGraph, Multimodal AI | Prototype |
| 7 | Intelligent Medical Data De-Identification System | AI-powered system for automated de-identification of medical records | MCP, LLM | Prototype |
| 8 | Medical Record Writing Guideline Q&A Chatbot | Q&A chatbot for institutional medical record documentation standards | MCP, RAG | Prototype |
| 9 | Voice-of-Customer Analysis Chatbot | Chatbot for analyzing patient feedback data with PostgreSQL integration | MCP, RAG, PostgreSQL | Prototype |
| 10 | Dataset Management System by LLM | LLM-based system for managing and querying research datasets | MCP, LLM | Prototype |
| 11 | Natural Language Search for Program Knowledge Management (AMIS 3.0) | Natural language search interface for the hospital’s program knowledge management system | MCP, RAG | Prototype |
| 12 | AGS Operational Guideline Q&A Chatbot | Natural-language Q&A chatbot for institutional operational guidelines (AGS), with original document viewer and text-to-speech (TTS); local LLM deployment for data security | MCP, RAG, qwen3-vl (local LLM) | Prototype |

Note: All projects used MCP as the core integration framework, consistent with the curriculum’s emphasis on agent-level architecture. One project (the Hospital Document Knowledge Retrieval RAG System) has been developed into an institutional service (“AI-Docs”) and is in active pilot use across multiple departments, including the Department of Nursing and the Big Data Research Center, with hospital-wide deployment planned within 2026; WorksBot received the Minister of Health and Welfare Award but is not yet in operational service. AGS (Asan Global Standard) refers to the institution's internal standards framework for continuous improvement of patient safety and healthcare quality, comprising structured standards (each with an intent statement) and measurable elements (MEs) that operationalize the actions required to meet them. AGS (Asan Global Standard) refers to the institution’s internal standards framework for continuous improvement of patient safety and healthcare quality, comprising structured standards (each with an intent statement) and measurable elements (MEs) that operationalize the actions required to meet them.
