# Supplementary material for: Teaching Model Context Protocol, Retrieval-Augmented Generation, and AI Agents to a Multidisciplinary Hospital Workforce: Single-Group Pre-Post Survey Study
Source: JMIR Med Educ. 2026 Sep 11;12:e97822. doi: 10.2196/97822 (PMC13568689; doi:10.2196/97822)
Supplement: Supplementary file 2 [file mededu-v12-e97822-s002.docx]

**Program:** Intensive Generative AI Training for a Multidisciplinary Hospital Workforce (“Advanced Course”, Medical AI Job-Competency Training Program, national initiative administered by the Korea Human Resource Development Institute for Health & Welfare)

**Duration:** 8 weeks (56 hours total = 31 hours on-site instruction + 25 hours self-directed capstone)

**Delivery:** On-site at Asan Medical Center with simultaneous online broadcast to two regional affiliate hospitals

**Tools provided:** Cursor IDE, Claude API, GPT API (funded subscriptions); dedicated internal cloud for local-LLM/MCP development

**Week 1 — 7 hours**

**Session 1-AM (3h, Lecture) — Foundation Models Overview**

*Learning objectives: Describe the evolution of AI and deep learning; explain foundation-model architectures and their relevance to healthcare applications.*

Topics / hands-on: AI history, deep-learning fundamentals, foundation-model architectures; course orientation.

**Session 1-PM (4h, Hands-on) — Prompt Engineering & Development Environment Setup**

*Learning objectives: Apply healthcare domain-specific prompting techniques; set up a working AI development environment.*

Topics / hands-on: Cursor IDE installation and configuration; structured prompting exercises on clinical/administrative tasks.

Tools: Cursor IDE

**Week 2 — 7 hours**

**Session 2-AM (3h, Hands-on) — Medical Document Q&A System**

*Learning objectives: Build a retrieval-augmented Q&A pipeline over medical documents; experience “vibe coding”.*

Topics / hands-on: PDF chunking, embedding, and RAG implementation; first-time coding via Cursor (vibe coding).; exercises were differentiated by track (application: a simple RAG pipeline in LangChain; development: implementing embedding and similarity search directly)

Tools: Cursor, vector store, LLM API

**Session 2-PM (4h, Hands-on) — LangGraph Workflow Design**

*Learning objectives: Model a clinical process as a graph-based workflow.*

Topics / hands-on: Graph-based design patterns; local-LLM (Ollama) integration; clinical process modeling.

Tools: LangGraph, Ollama

**Week 3 — 7 hours**

**Session 3-AM (3h, Lecture + Hands-on) — MCP Introduction & Healthcare Applications**

*Learning objectives: Explain the MCP architecture (Tools / Resources / Prompts) and identify healthcare use cases.*

Topics / hands-on: MCP core concepts; mapping medical use cases to MCP components.

**Session 3-PM (4h, Hands-on) — Vector Databases & RAG Agents**

*Learning objectives: Implement a RAG agent backed by a vector database.*

Topics / hands-on: LLM concepts for RAG; RAG-agent implementation; LangGraph-based agent development.

Tools: Vector DB, LangGraph

**Week 4 — 7 hours**

**Session 4-AM (3h, Lecture + Hands-on) — AI Agent Architecture & Design Principles**

*Learning objectives: Select agent types appropriate for clinical workflows and design for safety and explainability.*

Topics / hands-on: Agent types for medical workflows; safety and explainability; agent-architecture diagramming; identifying required tools and competencies; selection of each team's capstone service scenario.

**Session 4-PM (4h, Hands-on) — MCP Server Development & Team Strategy**

*Learning objectives: Build a basic MCP server; define a capstone team strategy.*

Topics / hands-on: LangChain/LangGraph frameworks; AI-agent architecture; capstone planning (team formation, problem selection).; differentiated by track (application: service-scenario concretization and MCP-tool integration; development: framework-based agent implementation with Ollama)

Tools: LangChain, LangGraph, MCP

**Weeks 5–7 — 25 hours (Self-directed, Team Capstone Projects I–III)**

**Mixed-expertise teams; 3–6 hours/week dedicated instructor mentoring per team**

*Learning objectives: Apply learned competencies to a real hospital problem; deliver a functional prototype.*

Topics / hands-on: Week 5 (I): MVP development — core functionality. Week 6 (II): refinement — enhancement and feature extension. Week 7 (III): quality assurance — stability hardening and quality evaluation. Deliverable: a functional MCP-based AI prototype addressing a real hospital problem (see Multimedia Appendix 2, Table S8 for the 12 projects).

**Week 8 — 7 hours**

**Session 8-AM (3h, Hands-on) — MCP Host Development & Workflow Integration**

*Learning objectives: Integrate MCP servers into a host application and orchestrate a medical workflow.*

Topics / hands-on: MCP host development; advanced LangGraph patterns; medical workflow orchestration.

**Session 8-PM-1 (1h, Lecture) — Institutional AI Services Overview**

*Learning objectives: Situate the program within current and future AI applications at the institution.*

Topics / hands-on: Current and future AI applications at Asan Medical Center.

**Session 8-PM-2 (3h, Presentation) — Capstone Presentations, Awards & Closing Ceremony**

*Learning objectives: Present capstone prototypes; receive panel evaluation.*

Topics / hands-on: 11 of 12 teams presented functional prototypes; evaluation by a panel of seven assessors using a standardized 100-point rubric (see Multimedia Appendix 4); the top-ranked project received the Minister of Health and Welfare Award.

Capstone Scenario Archetypes and Reusable MCP Modules

Capstone teams selected from six healthcare data-integration scenario archetypes that framed the curriculum's worked examples: (1) cross-hospital medical-record exchange (FHIR-based); (2) insurance-document generation (CDA-based); (3) clinical-research data extraction (CRF/eCRF); (4) de-identified data provision for external research; (5) mobile and wearable health-data integration (FHIR-based); and (6) a multi-institution integration platform.

These scenarios were supported by reusable MCP modules taught and reused across teams—Foundation modules: data retriever, code mapper, de-identifier, quality validator, clinical-context analyzer, and temporal aligner; Specialized modules: FHIR builder, CDA builder, CRF extractor, and synthetic-data generator. Hands-on practice used de-identified or synthetic datasets (e.g., MIMIC-derived FHIR resources) within the institution's secure internal cloud.

**Disclosure of Non-Released Materials**

To support reproducibility while respecting institutional and funding constraints, we release this detailed session-level syllabus and the capstone evaluation rubric (Multimedia Appendix 4). Full scaffold code, proprietary MCP server templates, and internal datasets are not released owing to institutional intellectual-property policy and national-program (Korea Human Resource Development Institute for Health & Welfare) constraints.
