# Supplementary material for: Teaching Model Context Protocol, Retrieval-Augmented Generation, and AI Agents to a Multidisciplinary Hospital Workforce: Single-Group Pre-Post Survey Study
Source: JMIR Med Educ. 2026 Sep 11;12:e97822. doi: 10.2196/97822 (PMC13568689; doi:10.2196/97822)
Supplement: Supplementary file 3 [file mededu-v12-e97822-s003.docx]

Capstone projects were evaluated at the closing ceremony (November 11, 2025) by a panel of seven assessors. Each of the 10 items was rated on a 1–10 scale (maximum 100 points). To adjust for differences in scoring stringency across assessors, each assessor’s raw scores were standardized (z-transformed) within assessor, converted to rank-based scores, and aggregated across the seven assessors to determine final standings. The top-ranked project received the Minister of Health and Welfare Award.

| Domain (points) | Items (each rated 1–10) | Points |
| --- | --- | --- |
| AI technical utilization | (1) AI clearly delivers the service’s core function; (2) AI’s role, scope, and contribution are clearly defined and identifiable; (3) appropriate and efficient methods are selected and applied; (4) creative methods are selected and applied | 40 (4 items) |
| User experience (UX/UI) | (1) Intuitiveness — users grasp the value immediately and use it with little learning; (2) Satisfaction — the experience is satisfying and positive | 20 (2 items) |
| Completeness & stability | (1) Core functions operate reliably without error as designed; (2) Exception handling and response speed are appropriate | 20 (2 items) |
| Clinical applicability | (1) Functions/services needed for clinical service and research support are designed; (2) Patient-data security and privacy protection are adequately reflected | 20 (2 items) |
| Total | 10 items | **100** |
