# Supplementary material for: Teaching Model Context Protocol, Retrieval-Augmented Generation, and AI Agents to a Multidisciplinary Hospital Workforce: Single-Group Pre-Post Survey Study
Source: JMIR Med Educ. 2026 Sep 11;12:e97822. doi: 10.2196/97822 (PMC13568689; doi:10.2196/97822)
Supplement: Supplementary file 4 [file mededu-v12-e97822-s004.docx]

This appendix presents English translations of all 64 free-text responses to the post-training open-ended question: “What aspect of change or growth did you most strongly feel through this 8-week program?” The responses were originally written in Korean and were translated into English for transparency and reproducibility. They are reproduced verbatim in anonymized form, with no personal identifiers, in arbitrary order. Minor typographical errors in the original responses were corrected in translation; content was not otherwise altered.

| No. | Response (English translation) |
| --- | --- |
| 1 | I came to clearly understand the conceptual aspects. |
| 2 | I got to apply what I learned in the training to research I am actually conducting. |
| 3 | Understanding of various concepts and hands-on practice. |
| 4 | I developed the will to take on the challenge. |
| 5 | I learned more concrete implementation methods. Because I now understand the concepts, I can run vibe coding and debug it. |
| 6 | Claude Code!!! Using RAG. |
| 7 | Confidence in using AI. |
| 8 | I came to clearly understand the concept of RAG. |
| 9 | I came to clearly understand the concepts and gained confidence in applying them. |
| 10 | AI, which had felt vague, became a little more familiar, and I developed the will to learn more. |
| 11 | Once I understood the concepts, my vague fear was resolved to some extent. |
| 12 | Understanding MCP was the most helpful to me. |
| 13 | Rising expectation rather than fear. |
| 14 | My fear of AI tools disappeared. |
| 15 | I got a mischievous thought that I might be able to delegate even more tasks.... |
| 16 | I gained a grasp of AI concepts that had been vague, and the confidence that I can make use of it. |
| 17 | As a non-developer with no knowledge of coding, I got to experience vibe coding, and I began thinking about how I might apply it to my work. |
| 18 | . |
| 19 | Understanding AI development concepts. |
| 20 | Improved understanding of AI and MCP. |
| 21 | It was good that they provided subscriptions to Claude and Cursor, giving us the opportunity. |
| 22 | How to use AI. |
| 23 | Becoming familiar with the terminology. |
| 24 | An opportunity to try out things I had known about (e.g., Cursor). |
| 25 | Skills for managing unstructured data. |
| 26 | Understanding AI concepts. |
| 27 | Experience using Claude Code. |
| 28 | My use of the Cursor agent improved greatly, substantially increasing my coding efficiency. |
| 29 | I became able to attempt coding, which had once felt out of reach, without hesitation. |
| 30 | Because the material was covered comprehensively over a long period, I feel my depth of understanding has increased. |
| 31 | I came to think about improvements to the systems we currently operate. |
| 32 | My first real experience of vibe coding! I was truly grateful for the paid Cursor version. If it keeps being provided, I think I'll use it at work too~ |
| 33 | Making concepts concrete. |
| 34 | For one thing, my understanding of currently known AI tools increased. |
| 35 | Understanding AI agents. |
| 36 | I gained the confidence that if I try, I can do it. |
| 37 | . |
| 38 | I recognized the applicability of AI and the potential for growth alongside AI. |
| 39 | Though not specific, I think I came to sense the rough workflow in the field and the direction for applying AI in the medical domain. |
| 40 | A change in my perception of AI. |
| 41 | As my knowledge and grasp of the operating principles behind vague AI concepts accumulated, I became able to think about how to apply them. |
| 42 | Well, first of all, the concept of MCP came across clearly to me. Because it was genuinely helpful, I had no regrets about taking the class, but I did have some reservations about the team composition. |
| 43 | Vibe coding made my work easier. |
| 44 | I felt the potential of AI a great deal. |
| 45 | If the training had been delivered on an in-hospital, hospital-tailored AI platform, its usefulness would have been greater. |
| 46 | As I started vibe coding, I was able to understand more clearly the many terms and concepts I had only vaguely known, and I got a rough sense of the guidelines for building an LLM. |
| 47 | I used to distrust AI and could not delegate work to it, but now my perception of AI has changed to be much more positive. |
| 48 | MCP was really great. |
| 49 | My perception of AI improved a lot. And I gained some confidence too. |
| 50 | I came up with a few ideas worth incorporating. |
| 51 | I came to clearly understand the concept of RAG. |
| 52 | I came to clearly understand several concepts. |
| 53 | Improved overall understanding of AI. |
| 54 | I believe that for something new to advance, improving the perception of existing users is most important. In that sense, this training feels like another new beginning for me. I had only felt resistance toward AI, but the quality of the coding was higher and the speed faster than I expected, and I came to think that using AI agents could raise our technical capability further. I resolved not to fear the advance of AI but to apply it well. |
| 55 | None. |
| 56 | Understanding RAG. |
| 57 | My first goal was “recovering confidence,” and I commend myself for having fully achieved it. I want to actively use the parts on RAG and de-identification, and to provide know-how that also helps users. Thank you for the opportunity to learn, and you all worked really hard. ^^ |
| 58 | Exploring scenarios that use AI technology, application plans, and so on. |
| 59 | A rise in my understanding of AI. |
| 60 | How to use AI. |
| 61 | Increased understanding of AI and acquiring the foundational knowledge to apply it in the actual work environment. |
| 62 | Increased understanding of AI. |
| 63 | It was that I brought MCP and RAG, which had felt vague, into hospital work and implemented them as visible results through hands-on practice. Seeing it in lecture materials and doing it myself were truly different experiences. It was hard but rewarding, and I think it will stay with me for a long time. |
| 64 | AI awareness. |
