# Supplementary material for: Teaching Model Context Protocol, Retrieval-Augmented Generation, and AI Agents to a Multidisciplinary Hospital Workforce: Single-Group Pre-Post Survey Study
Source: JMIR Med Educ. 2026 Sep 11;12:e97822. doi: 10.2196/97822 (PMC13568689; doi:10.2196/97822)
Supplement: Supplementary file 5 [file mededu-v12-e97822-s005.docx]

# STROBE Statement — Checklist of Items for Cross-Sectional Studies

Supplementary Table. STROBE checklist mapping for the present study. Reference: von Elm E, et al. The STROBE statement. *Lancet*. 2007;370:1453-1457.

| # | Item | Recommendation | Reported in |
| --- | --- | --- | --- |
| **Title and abstract** |  |  |  |
| 1 | (a) Indicate the study’s design with a commonly used term in the title or the abstract | Cross-sectional (pre-post comparison) | Title, Abstract |
|  | (b) Provide in the abstract an informative and balanced summary of what was done and what was found | Structured abstract with Background, Objective, Methods, Results, Conclusions | Abstract |
| **Introduction** |  |  |  |
| 2 | Explain the scientific background and rationale for the investigation being reported | AI in healthcare, workforce readiness gap, literature gap in advanced AI training | Introduction, Background; Introduction, Prior Work |
| 3 | State specific objectives, including any prespecified hypotheses | Four research questions (RQ1–RQ4) with Kirkpatrick framework mapping; dual aims (curriculum description + outcome evaluation) | Introduction, Study Objectives |
| **Methods** |  |  |  |
| 4 | Present key elements of study design early in the paper | Single-group pre-post survey, independent samples design | Methods, Study Design |
| 5 | Describe the setting, locations, and relevant dates | Tertiary academic medical center in Seoul, South Korea; Sep–Nov 2025 | Methods, Study Design |
| 6 | (a) Give the eligibility criteria, and the sources and methods of selection of participants | Inclusion: current employees, voluntary enrollment, age ≥18; Exclusion: <80% attendance (all 83 met threshold) | Methods, Participants |
| 7 | Clearly define all outcomes, exposures, predictors, potential confounders, and effect modifiers | Primary (S4, S6), secondary (S2, S3, S5), post-only (S9, S10, S11) outcomes defined with item counts and Kirkpatrick mapping | Methods, Outcome Measures |
| 8 | For each variable of interest, give sources of data and details of methods of assessment (measurement) | 5-point Likert scale; survey developed by research team; Cronbach’s alpha reported | Methods, Outcome Measures |
| 9 | Describe any efforts to address potential sources of bias | Chi-squared tests for demographic comparability; worst-case sensitivity analysis for non-response bias (Supplementary Table S7); acknowledgment of self-selection and attrition bias | Methods, Statistical Analysis; Discussion, Limitations |
| 10 | Explain how the study size was arrived at | Convenience sample; sensitivity-style power analysis (minimum detectable r = .24 at 80% power) | Methods, Statistical Analysis |
| 11 | Explain how quantitative variables were handled in the analyses | Likert-scale data treated as ordinal; Mann-Whitney U tests; rank-biserial r and Cohen’s d | Methods, Statistical Analysis |
| 12 | (a) Describe all statistical methods | Mann-Whitney U, chi-squared, Cronbach’s alpha, rank-biserial r, Cohen’s d, EFA | Methods, Statistical Analysis |
|  | (b) Describe any methods used to examine subgroups and interactions | Subgroup analyses by professional group using Cohen’s d with 95% CIs; no multiple comparison correction (exploratory) | Methods, Statistical Analysis |
|  | (c) Explain how missing data were addressed | Anonymous survey; no individual-level matching possible; complete-case analysis | Methods, Study Design; Participants |
|  | (d) If applicable, describe analytical methods taking account of sampling strategy | N/A (convenience sample) | — |
|  | (e) Describe any sensitivity analyses | Sensitivity-style power analysis for minimum detectable effect size; worst-case non-response sensitivity analysis | Methods, Statistical Analysis |
| **Results** |  |  |  |
| 13 | (a) Report numbers of individuals at each stage of study | 83 enrolled → 83 completed program → 64 post-survey respondents (response rate: 77.1%) | Methods, Participants; Results, Participant Characteristics |
|  | (b) Give reasons for non-participation at each stage | 19 non-response to post-survey | Methods, Participants; Discussion, Limitations |
|  | (c) Consider use of a flow diagram | Participant flow diagram in Figure 4 | Results, Participant Characteristics |
| 14 | (a) Give characteristics of study participants and information on exposures and potential confounders | Table 1: demographic characteristics by pre/post group | Results, Participant Characteristics; Table 1 |
|  | (b) Indicate number of participants with missing data for each variable of interest | Complete responses required by survey design (all items mandatory) | Methods, Outcome Measures |
| 15 | Report numbers of outcome events or summary measures | Means, SDs, U statistics, Z values, p values, effect sizes for all comparisons; representative capstone projects as qualitative outcome examples (Table 4) | Results; Tables 2–4 |
| 16 | (a) Give unadjusted estimates and, if applicable, confounder-adjusted estimates and their precision | Unadjusted Mann-Whitney U with 95% CIs for subgroup Cohen’s d | Results; Table 3 |
|  | (b) Report category boundaries when continuous variables were categorized | S3 AI usage: categorical boundaries defined (< 1h, 1–3h, 3+ h daily) | Results, Secondary Outcomes (S3) |
|  | (c) If relevant, consider translating estimates of relative risk into absolute risk for a meaningful time period | N/A (not a risk study) | — |
| 17 | Report other analyses done — e.g., analyses of subgroups and interactions, and sensitivity analyses | Subgroup analysis by professional group (Table 3); sensitivity-style power analysis; worst-case non-response sensitivity analysis (Supplementary Table S7) | Results; Methods, Statistical Analysis |
| **Discussion** |  |  |  |
| 18 | Summarise key results with reference to study objectives | Five principal lessons aligned with RQ1–RQ4 | Discussion, Principal Findings |
| 19 | Discuss limitations of the study, taking into account sources of potential bias or imprecision | Study design constraints, attrition and selection bias, measurement limitations, generalizability and temporal scope | Discussion, Limitations |
| 20 | Give a cautious overall interpretation of results considering objectives, limitations, multiplicity of analyses, results from similar studies, and other relevant evidence | Comparison with prior work; five lessons with caveats; subgroup uncertainty noted; cross-sectional limitations acknowledged | Discussion, Principal Findings; Discussion, Comparison with Prior Work |
| 21 | Discuss the generalizability (external validity) of the study results | Single institution, self-selected participants; recommendations for future multi-site studies | Discussion, Limitations |
| 22 | Give the source of funding and the role of the funders | KHIDI grants HR21C0198 and RS-2025-02213531; Ministry of Health and Welfare, Republic of Korea | Funding Statement |
